# *APOE4* Exerts Partial Diet-dependent Effects on Energy Expenditure and Skeletal Muscle Mitochondrial Pathways in a Preclinical Model

**DOI:** 10.1093/function/zqaf017

**Published:** 2025-03-25

**Authors:** Chelsea N Johnson, Colton R Lysaker, Elaine C Gast, Colin S McCoin, Riley E Kemna, Kelly N Z Fuller, Benjamin A Kugler, Edziu Franczak, Vivien Csikos, Julie Allen, Casey S John, MaryJane A Wolf, Matthew E Morris, John P Thyfault, Heather M Wilkins, Paige C Geiger, Jill K Morris

**Affiliations:** Department of Cell Biology and Physiology, University of Kansas Medical Center, Kansas City, KS 66160, USA; University of Kansas Alzheimer's Disease Research Center, University of Kansas Medical Center, Fairway, KS 66205, USA; University of Kansas Alzheimer's Disease Research Center, University of Kansas Medical Center, Fairway, KS 66205, USA; Department of Cell Biology and Physiology, University of Kansas Medical Center, Kansas City, KS 66160, USA; Department of Cell Biology and Physiology, University of Kansas Medical Center, Kansas City, KS 66160, USA; University of Kansas Diabetes Institute, University of Kansas Medical Center, Kansas City, KS 66160, USA; University of Kansas Alzheimer's Disease Research Center, University of Kansas Medical Center, Fairway, KS 66205, USA; University of Colorado Anschutz Medical Campus, Aurora, CO 80045, USA; Department of Cell Biology and Physiology, University of Kansas Medical Center, Kansas City, KS 66160, USA; Department of Cell Biology and Physiology, University of Kansas Medical Center, Kansas City, KS 66160, USA; University of Kansas Alzheimer's Disease Research Center, University of Kansas Medical Center, Fairway, KS 66205, USA; Department of Cell Biology and Physiology, University of Kansas Medical Center, Kansas City, KS 66160, USA; University of Kansas Alzheimer's Disease Research Center, University of Kansas Medical Center, Fairway, KS 66205, USA; Department of Cell Biology and Physiology, University of Kansas Medical Center, Kansas City, KS 66160, USA; Department of Cell Biology and Physiology, University of Kansas Medical Center, Kansas City, KS 66160, USA; University of Kansas Diabetes Institute, University of Kansas Medical Center, Kansas City, KS 66160, USA; Department of Cell Biology and Physiology, University of Kansas Medical Center, Kansas City, KS 66160, USA; University of Kansas Alzheimer's Disease Research Center, University of Kansas Medical Center, Fairway, KS 66205, USA; University of Kansas Diabetes Institute, University of Kansas Medical Center, Kansas City, KS 66160, USA; University of Kansas Alzheimer's Disease Research Center, University of Kansas Medical Center, Fairway, KS 66205, USA; Department of Neurology, University of Kansas Medical Center, Kansas City, KS 66160, USA; Department of Cell Biology and Physiology, University of Kansas Medical Center, Kansas City, KS 66160, USA; University of Kansas Diabetes Institute, University of Kansas Medical Center, Kansas City, KS 66160, USA; University of Kansas Alzheimer's Disease Research Center, University of Kansas Medical Center, Fairway, KS 66205, USA; Department of Neurology, University of Kansas Medical Center, Kansas City, KS 66160, USA

**Keywords:** *APOE4*, Alzheimer's, mice, whole-body metabolism, skeletal muscle, proteomics, mitochondria

## Abstract

Apolipoprotein E4 (*APOE4*) is the greatest genetic risk factor for Alzheimer's (AD) and is linked to whole-body metabolic dysfunction. However, it is unclear how *APOE4* interacts with modifiable factors like diet to impact tissues central to regulating whole-body metabolism. We examined *APOE4-* and Western diet-driven effects in skeletal muscle using *APOE3* (control) and *APOE4* targeted replacement mice on a C57BL/6NTac background fed a high-fat diet (HFD, 45% kcal fat) or low-fat diet (LFD, 10% kcal fat) for 4 months (*n* = 7-8 per genotype/diet/sex combination). We assessed body composition and whole-body outcomes linked to skeletal muscle function including respiratory exchange ratio (RER) and resting energy expenditure (REE). In skeletal muscle, we evaluated the proteome and mitochondrial respiration. In males only, *APOE4* drove greater gains in fat mass and lower gains in lean mass on both diets. *APOE4* did not affect daily RER but was associated with elevated REE in males and lower REE in HFD females after covarying for body composition. Skeletal muscle proteomics showed *APOE4* exerts several diet- and sex-specific effects on mitochondrial pathways, including elevations in branched-chain amino catabolism in HFD males and reductions in oxidative phosphorylation in LFD females. This did not translate to differences in skeletal muscle mitochondrial respiration, suggesting that compensatory mechanisms may sustain mitochondrial function at this age. Our work indicates that genetic risk may mediate early life effects on skeletal muscle mitochondria and energy expenditure that are partially dependent on diet. This has important implications for mitigating ad risk in *APOE4* carriers.

## Graphical Abstract

**Graphical Abstract:**
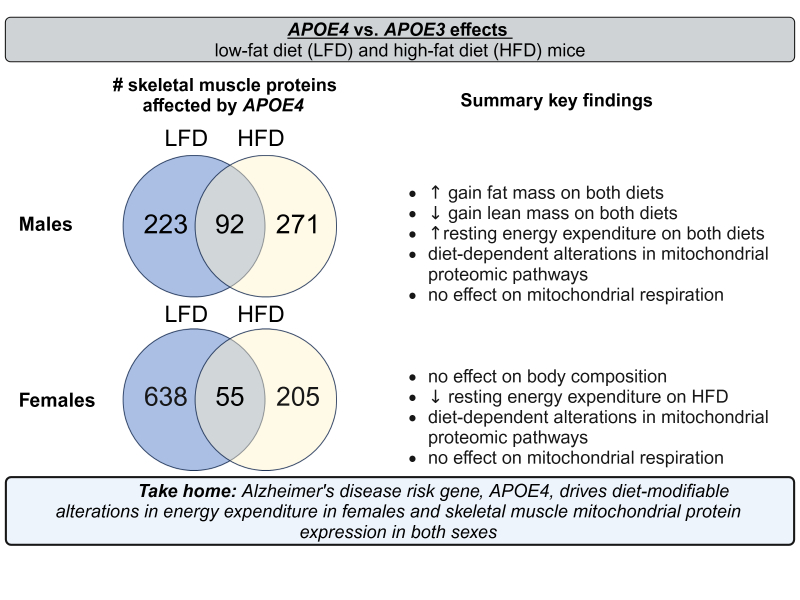

## Introduction

Alzheimer’s disease (AD) affects approximately 6.9 million Americans, and this number continues to grow.^1^ Apolipoprotein E4 (*APOE4*), 1 of 3 isoforms that encode variants of the lipid transport protein APOE, is the greatest genetic risk factor for AD. The prevalence of AD is approximately 20% in individuals without an *APOE4* allele compared to 47% with 1 *APOE4* copy and 91% with 2 *APOE4* copies, highlighting the significant risk imparted by this allele.^2^ The other 2 isoforms, *APOE2* and *APOE3*, reduce or do not affect risk, respectively. Although it is unclear how *APOE4* mediates AD risk, increasing evidence supports a role for bioenergetic dysfunction. Individuals with AD display early reductions in brain glucose metabolism,^3^ which is further diminished in *APOE4* carriers.^4^ *APOE4* is also linked to peripheral defects in metabolism including increased LDL cholesterol and elevated risk of cardiovascular disease.^5^ Diet impacts similar aspects of lipid metabolism^6^ and observational studies indicate that a diet high in saturated fats increases vulnerability to cognitive impairment.^7^ Additionally, shifts in diet may have driven the evolution of different *APOE* isoforms from the ancestral *APOE4* allele.^8^ However, little is known about the interaction between *APOE4* and diet in tissues that may mediate the relationship between whole-body metabolic function and brain health.

Skeletal muscle is an important determinant of whole-body basal metabolic rate, oxygen consumption, insulin-stimulated glucose uptake, and substrate preference.^9–12^ While its role in ad progression is not fully understood, skeletal muscle is affected in early disease. Muscle strength and motor function are reduced during preclinical AD and lean mass positively correlates with cognitive function during the initial phases of clinical ad, supporting a relationship between muscle and brain health.^13–16^ Further, the presence of an *APOE4* allele is associated with greater reductions in motor function over time among cognitively healthy older individuals.^17^ We have shown that during early stages of cognitive decline, lipid-supported mitochondrial respiration is reduced in skeletal muscle biopsy tissue.^18^ We also recently found that *APOE4* alters the expression of mitochondrial pathways that are directly involved in energy metabolism in skeletal muscle of 4-month-old mice, which corresponds to a young adult human.^19^ Despite these findings, it's unclear how *APOE4* may interact with modifiable factors like diet to influence skeletal muscle and whole-body metabolism.

Using *APOE4* and *APOE3* (control) targeted replacement (TR) mice fed a low-fat diet (LFD) or high-fat diet (HFD), we investigated the interaction between *APOE* genotype and diet on skeletal muscle and whole-body metabolic outcomes. Since female sex is associated with faster reductions in cerebral glucose metabolism relative to males and lower energy expenditure when carrying *APOE4*,^20,21^ we considered metabolic differences within males and females separately. Our hypothesis was that *APOE4* and HFD would induce similar, compounding effects on metabolism, resulting in HFD *APOE4* TR mice exhibiting the most pronounced metabolic derangements. Here, we provide evidence that *APOE4* drives unfavorable changes in body composition and elevations in energy expenditure in male mice, regardless of diet. *APOE4* also mediates HFD-dependent reductions in energy expenditure in females and changes in the skeletal muscle proteome of male and female mice. While pathways supporting mitochondrial metabolism are generally upregulated by *APOE4* in male skeletal muscle and downregulated by *APOE4* in female skeletal muscle, these effects are modified by diet (eg, branched-chain amino catabolism is only upregulated in HFD males while oxidative phosphorylation is only downregulated in LFD females). Our findings suggest that diet may be an important tool to modulate energy expenditure and skeletal muscle mitochondrial pathways affected by *APOE4*.

## Materials and Methods

### Animals

Male and female *APOE3* and *APOE4* TR mice (*n* = 61 total, 7-8 per genotype/diet/sex combination) purchased from Taconic were housed in groups of 3-4 and maintained on a 14-hour light/10-hour dark cycle. Mice were fed a standard chow diet (14% kcal fat, Teklad Global Rodent Diets, 8604) until 4 months old, when mice were switched to a LFD (D12110704, 10% kcal fat, Research Diets, New Brunswick, NJ) or HFD (D12451, 45% kcal fat, Research Diets) for 4 months. At 8 months old, mice were anesthetized with phenobarbital (0.5 mg phenobarbital/g body weight) prior to tissue harvest. All experiments in living animals were approved by the Institutional Animal Care and Use Committee at the University of Kansas Medical Center.

### Body Anthropometrics and Indirect Calorimetry

Body mass, body composition, energy expenditure, and respiratory exchange ratio were measured at 4 months old before diet start and again before sacrifice at 8 months old. Body composition was analyzed on an EchoMRI TM 1100 Analyzer (EchoMRI, Houston, TX, USA). Mice were single-housed in the Promethion Indirect Calorimetry system (Sable Systems International, Las Vegas, NV, USA) for 7 days total to assess respiratory exchange ratio (RER) and total (TEE), resting (REE), and non-resting (NREE) energy expenditure. Mice were acclimated to the cages for 3 days prior to data collection. Average daily TEE, REE, NREE, and RER were calculated from days 4 and 5. This was further split into dark (more active) and light (less active) cycles. At the end of day 5, mice were challenged with a 16-hour fast followed by a 24-hour refeeding period to assess metabolic flexibility. Food consumption and activity were tracked while in the cages.

### Glucose Tolerance Test

Mice were fasted for 4 hours prior to glucose tolerance testing (GTT) at 7 months old. Blood glucose was measured in tail blood on a Clarity BG1000 blood glucose monitoring system (Clarity diagnostics, Boca Raton, FL, USA) at baseline and 15, 30, 45, 60, 90, and 120 min after bolus intraperitoneal injection of 1 g glucose/kg lean mass. Values greater than the maximum reading of the glucometer (600 mg/dl) were recorded as 600 and retained for analysis to limit biased weighting of lower glucose values.

### Tissue Collection

Cardiac blood was collected in an Eppendorf tube and centrifuged at 7000 *g* for 10 min at 4°C. The supernatant was transferred to a new tube and stored at −80°C to assess serum lactate. The right quadriceps was immediately cleared of fat and connective tissue and placed in ice-cold mitochondrial isolation buffer (220 m m mannitol, 70 m m sucrose, 10 m m Tris,1 m m EDTA, pH 7.4) prior to processing for respirometry analyses. The left quadriceps was cleared of fat and connective tissue, embedded in OCT, then stored at −80°C for tissue staining. The left gastrocnemius was cleared of fat and connective tissue and stored at −80°C for proteomics.

### Serum Lactate

L-lactate was measured in serum processed at the time of sacrifice using a colorimetric kit according to the manufacturer's instructions (Abcam ab65331, Cambridge, MA, USA).

### Protein Isolation From Skeletal Muscle

Gastrocnemius tissue was finely crushed with a pestle and placed in a tube cooled in liquid nitrogen. Homogenization buffer (1% Triton X-100, 50 m m HEPES, 12 m m Na pyrophosphate, 100 m m NaF, 10 m m EDTA, protease inhibitor, phosphatase inhibitor) and a stainless-steel bead were immediately added to the crushed tissue and samples were homogenized on a TissueLyser II (Qiagen, Germantown, MD). Samples were beat 3 times at 20 Hz for 2 min and placed on ice for 10 min between sets. Following homogenization, samples were centrifuged at 15 000 *g* for 25 min at 4°C and the supernatant containing the lysed proteins were transferred to a new tube. A bicinchoninic acid assay (Thermo Fisher, Waltham, MA, USA) was used to measure protein content and 50 μg of protein were sent to the University of Arkansas for Medical Sciences (UAMS) for proteomics.

### Skeletal Muscle Proteomics

Gastrocnemius protein lysates were processed for data-independent acquisition mass spectrometry at UAMS as previously described.^22^ Briefly, proteins were reduced, alkylated, extracted with chloroform/methanol, and digested into peptides with porcine trypsin (Promega). Peptides were separated by reverse phase XSelect CSH C18 2.5 um resin (Waters) on an in-line 150 × 0.075 mm column using an UltiMate 3000 RSLCnano system (Thermo) and ionized by electrospray (2.2 kV) before analysis on an Orbitrap Exploris 480 mass spectrometer (Thermo). Spectronaut (Biognosys version 18.3) was used to search data against the UniProt Mus musculus database (September 2023).

### Mitochondrial Isolation From Skeletal Muscle

Quadriceps tissue was placed in ice-cold mitochondrial isolation buffer (220 m m mannitol, 70 m m sucrose, 10 m m Tris,1 m m EDTA, pH 7.4) prior to being minced and digested with 2.5 μL trypsin (0.05%) for 30 min. Trypsin was removed by centrifugation (4°C, 3 min, 200 *g*). The pellet was then resuspended in 13 mL of isolation buffer with 0.1% BSA and homogenized on ice using a glass-on-glass homogenizer and transferred to a new tube. The homogenized tissue was centrifuged (4°C, 10 min, 700 *g*) and the resulting supernatant was transferred to a new tube and centrifuged (4°C, 10 min, 8000 *g*). The pellet was resuspended in 5 mL of isolation buffer and centrifuged again (4°C, 10 min, 8000 *g*). The final pellet was resuspended in 200 μL of respiration buffer (0.5 m m EGTA, 3 m m MgCl_2_, 60 m m KMES, 20 m m glucose,10 m m KH_2_PO4, 20 m m HEPES, 110 m m sucrose, 0.1% BSA, pH 7.1) and protein concentration was measured using a bicinchoninic acid assay (Thermo Fisher).

### Respirometry in Skeletal Muscle Mitochondrial Isolates

We first used standard methods to measure O_2_ flux in mitochondria isolated from the quadriceps on the Oroboros Oxygraph-2k system (Innsbruck, Austria). H_2_O_2_ was simultaneously measured as previously described.^23^ For carbohydrate-supported respiration, basal oxygen consumption was measured at steady state in the presence of 2 m m malate, 0.01 m m CoA, 2.5 m m carnitine, and 5 m m potassium pyruvate. Complex I-linked respiration was assessed by adding 2.5 m m ADP (state 3) followed by 2 m m glutamate (state 3 + glutamate). Lastly, 10 m m succinate was added to measure complex II-linked respiration (state 3S). For lipid-supported respiration, basal oxygen consumption was measured in the presence of 2 m m malate, 0.01 m m CoA, 2.5 m m carnitine, and 0.01 m m palmitoyl-CoA. After reaching steady state, 2.5 m m ADP was added to assess state 3 respiration. This was followed by 0.01 m m palmitoyl-carnitine (PC) (state 3 + PC) to measure state 3 respiration unrestricted by the rate-limiting enzyme, carnitine palmitoyltransferase 1 (state 3 + PC). After achieving steady state, 10 m m succinate was added to interrogate maximal complex II-linked respiration (state 3S). All O_2_ flux and H_2_O_2_ flux values were normalized to protein content.

We also used a modified form of the creatine kinase (CK) clamp assay to assess carbohydrate-supported respiration under a range of physiological ATP:ADP ratios to model in vivo changes in energetic demand.^24^ First, oxygen consumption was measured in the presence of 0.01 m m CoA, 2.5 m m carnitine, 5 m m creatine monohydrate, 2 m m malate, 5 m m potassium pyruvate, 20 m m creatine kinase, 5 m m ATP, and 1 m m PCr. PCr was then added in steps to assess oxygen flux at the following PCr concentrations: 3 m m, 6 m m, 9 m m, 12 m m, 15 m m, 18 m m, and 21 m m. The free energy of ATP hydrolysis (-∆G_ATP_) was calculated using a web-based calculator (https://dmpio.github.io/bioenergetic-calculators/ck_clamp/) developed by Fisher-Wellman, et al.^25^ Respiratory conductance was determined by calculating the slope of the relationship between O_2_ flux and ∆G_ATP_ within the linear range. O_2_ values measured outside the linear range were not reported.

### Tissue Staining

Serial quadriceps cross-sections were cut 10 µm thick. For fiber-typing, sections were hydrated with PBST for 5 min, blocked for 1 hour, washed 3 times with PBST, then stained 1:100 for primary antibodies against type 1 (BA-F8 mIgG_2_b), type IIa (SC-71 mIgG_1_), and type IIb (BF-F3 mIgM) fibers all purchased from DSHB (Iowa City, IA) and 1:200 for laminin (Abcam ab11575) overnight. Sections were then washed 3 times with PBST, stained 1:200 for all secondary antibodies purchased from Invitrogen for 1 hour (mIgG_2_b-AF350 A21140, mIgG_1_-AF488 A21121, mIgM-AF555 A21426, rbIgG-AF647 A21244), washed 3 times with PBST, mounted, and immediately imaged. Unstained fibers represented type IIx fibers. All images were acquired on a Zeiss Observer 7 microscope (Carl Zeiss Microscopy, White Plains, NY) and analyzed for fiber-type distribution using MuscleJ2.^26^ To visually assess lipid droplets, freshly cut sections were immediately fixed in 4% paraformaldehyde for 1 hour. Following fixation, tissues were washed 3 times with PBS, stained overnight with 1:200 laminin primary antibody (Abcam ab11575, Cambridge, MA), and washed 3 times with PBS. To visualize laminin, sections were incubated in 1:200 rbIgG antibody (Invitrogen A21244, Carlsbad, CA) for 1 hour and washed 3 times with PBS. Sections were then incubated in 0.1 µg/ml BODIPY 493/503 (Invitrogen D3922) for 30 minutes, washed 3 times with PBS, mounted, and immediately imaged on a Zeiss Observer 7 microscope (Carl Zeiss Microscopy).

### Statistical Analyses

#### Statistical Analysis of Proteomic Data

Proteomics data was processed at UAMS with variance stabilization normalization.^27^ ProteoDA was used for statistical analysis using Linear Models for Microarray Data (limma) with empirical Bayes (eBayes) smoothing to standard errors.^28,29^ Proteins with a *P* ≤ 0.05 for between group differences were considered significant. Ingenuity pathway analysis (IPA, Quiagen) was used to assess for pathway enrichment (*P*-value of overlap) and predicted direction of pathway activity (activation z-score) based on the analysis of proteins that significantly differed between groups. Enrichment *P*-values ≤ 0.05 and absolute z-score values ≥ 2 were considered statistically significant. All pathways with a reported z-score had a significant enrichment p-value. For each sample, the summed intensity of mitochondrial proteins, determined with MitoCarta3.0,^30^ was divided by the summed intensity of all proteins and multiplied by 100 to compare mitochondrial protein abundance between samples.

#### Statistical Analysis of Remaining Data

Analysis of remaining data was performed in SPSS 29.0. All analyses were split by sex. Boxplot inspection was used to identify and exclude outliers that were greater than 3 box-lengths from the edge of the box. An independent sample *t*-test was used to analyze the effect of *APOE* genotype before diet start. For data involving 2 between-subjects factors (*APOE* genotype and diet), 2-way ANOVA was used to assess for an interaction between these factors. For indirect calorimetry data involving fat mass and lean mass as covariates,^31^ ANCOVA was used to compare estimated marginal means between groups. For ANOVA and ANCOVA analyses, main effects of *APOE* genotype and diet were interpreted if the interaction was not significant. Significant interactions were followed up with multiple comparisons using Fisher's Least Significance Difference (LSD). For glucose tolerance data involving 2 between-subjects factors (genotype and diet) and 1 within subjects’ factor (time), 3-way mixed ANOVA was used to assess the interaction between these 3 factors. Two-way interactions were interpreted if the 3-way interaction was not significant and main effects were interpreted if the 2-way interaction was not significant. Glucose AUC was calculated from both raw and baseline (fasting) corrected values using the trapezoidal method. Baseline corrections were performed by subtracting the starting glucose value from the glucose value measured at each timepoint,^32^ Group differences in glucose AUC were assessed with a 2-way ANOVA. The significance threshold for all tests was *P* ≤ 0.05.

## Results

### Whole-body Metabolic Characterization of *APOE3* and *APOE4* TR Mice on LFD or HFD

#### Influence of APOE4 and HFD on Body Mass and Composition

**Figure 1.**
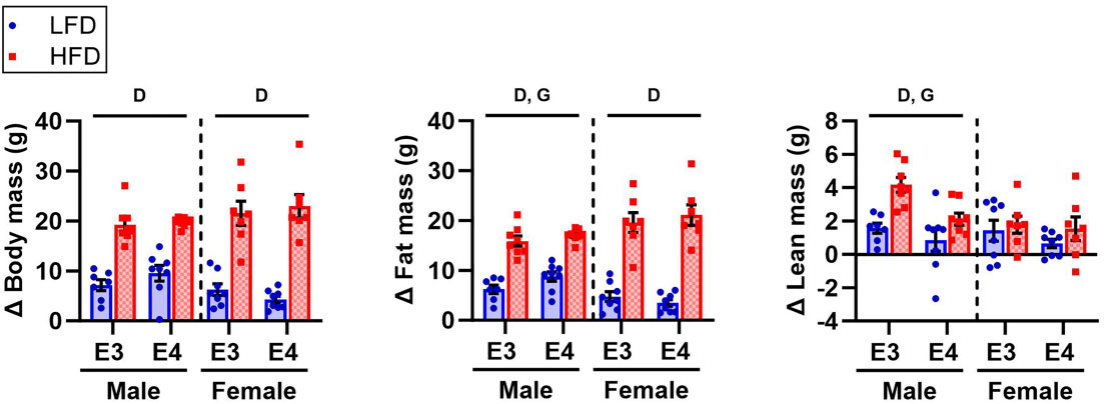
Impact of *APOE4* and HFD on body composition. Body mass and composition were assessed at 4 months old and prior to sacrifice at 8 months old after 4 months of diet. Change in body mass, fat mass, and lean mass after 4 months of diet are shown. Sample size: *n* = 7-8/group. The following symbols represent comparisons with *P* ≤ 0.05: D, main effect diet; G, main effect genotype. LFD, low-fat diet; HFD, high-fat diet; E3, apolipoprotein E3; E4, apolipoprotein E4.

Body anthropometrics were determined prior to diet start and after 4 months of diet. Before diet, there were no significant differences in body mass or composition between *APOE3* and *APOE4* TR mice (Table S1). After 4 months of diet, male *APOE4* TR mice gained more fat mass and less lean mass than male *APOE3* TR mice (Figure 1). This corresponded with male *APOE4* TR mice having greater body and fat mass and lower % lean mass than male *APOE3* TR mice on both diets at 8 months old (Table 1). In females, lean mass trended lower at 8 months in *APOE4* versus *APOE3* TR mice (*P* = 0.066) on both diets (Table 1). Diet significantly impacted body composition in males and females. HFD mice gained more body and fat mass compared to LFD mice regardless of sex or *APOE* genotype (Figure 1, Table 1). While HFD was also associated with a larger increase in lean mass in males, % lean mass was lower and % fat mass was higher in HFD versus LFD mice after 4 months of diet (Figure 1, Table 1).

**Table 1.** Influence of *APOE4* and HFD on body composition.

Males							
8 Months (4 months post-diet)							
	*APOE3*		*APOE4*		ANOVA *P*-values		
	LFD	HFD	LFD	HFD	Diet × genotype interaction	Diet main effect	Genotype main effect
Body mass (g)	35.0 [4.19]	47.8 [3.40]	39.6 [4.19]	48.8 [1.97]	0.176	<0.001*	0.037*
Fat mass (g)	9.10 [3.08]	19.86 [2.41]	12.42 [3.48]	20.64 [0.76]	0.192	<0.001*	0.039*
Fat mass (%)	25.41 [6.74]	41.47 [2.95]	30.83 [6.48]	42.33 [1.89]	0.208	<0.001*	0.087
Lean mass (g)	24.69 [1.93]	26.86 [2.25]	25.10 [1.19]	25.89 [1.99]	0.314	0.038*	0.682
Lean mass (%)	71.06 [7.38]	56.31 [4.54]	63.90 [6.05]	52.98 [2.26]	0.325	<0.001*	0.011*
Change 4 to 8 Months							
	*APOE3*		*APOE4*		ANOVA *P*-values		
	LFD	HFD	LFD	HFD	Diet × genotype interaction	Diet main effect	Genotype main effect
∆ Body mass (g)	7.2 [2.90]	19.3 [3.66]	9.6 [4.39]	19.8 [0.95]	0.432	<0.001*	0.227
∆ Fat mass (g)	6.21 [2.25]	15.88 [2.88]	8.77 [2.74]	17.07 [1.23]	0.427	<0.001*	0.037*
∆ Fat mass (%)	15.12 [4.11]	27.70 [5.04]	18.80 [4.43]	29.95 [3.00]	0.642	<0.001*	0.062
∆ Lean mass (g)	1.56 [0.80]	4.17 [1.25]	0.86 [1.88]	2.12 [1.01]	0.166	<0.001*	0.007*
∆ Lean mass (%)	−12.01 [3.73]	−23.42 [5.40]	−16.81 [3.57]	−28.97 [3.52]	0.802	<0.001*	0.002*
Females							
8 Months (4 months post-diet)							
	*APOE3*		*APOE4*		ANOVA p-values		
	LFD	HFD	LFD	HFD	Diet* genotype interaction	Diet main effect	Genotype main effect
Body mass (g)	30.6 [4.80]	46.1 [7.13]	27.5 [2.44]	46.2 [7.65]	0.449	<0.001*	0.477
Fat mass (g)	8.15 [3.47]	22.72 [5.69]	6.15 [2.41]	24.00 [5.67]	0.322	<0.001*	0.829
Fat mass (%)	25.64 [7.48]	48.60 [5.52]	21.93 [6.68]	51.47 [3.61]	0.151	<0.001*	0.853
Lean mass (g)	20.30 [1.58]	21.26 [1.42]	19.28 [0.67]	20.12 [2.21]	0.923	0.122	0.066
Lean mass (%)	67.17 [6.50]	46.70 [4.73]	70.57 [5.34]	43.90 [2.97]	0.110	<0.001*	0.874
Change 4 to 8 Months							
	*APOE3*		*APOE4*		ANOVA *P*-values		
	LFD	HFD	LFD	HFD	Diet* genotype interaction	Diet main effect	Genotype main effect
∆ Body mass (g)	6.3 [3.32]	21.6 [6.41]	4.3 [1.89]	22.9 [6.18]	0.338	<0.001*	0.857
∆ Fat mass (g)	4.77 [2.78]	19.61 [5.28]	3.45 [1.88]	21.09 [5.47]	0.353	<0.001*	0.954
∆ Fat mass (%)	11.97 [5.51]	36.00 [4.73]	10.42 [5.18]	38.93 [4.24]	0.228	<0.001*	0.707
∆ Lean mass (g)	1.42 [1.77]	1.78 [1.35]	0.65 [0.72]	1.53 [1.87]	0.635	0.264	0.353
∆ Lean mass (%)	−10.55 [4.81]	−32.68 [3.97]	−9.83 [4.06]	−35.95 [4.56]	0.223	<0.001*	0.435

* *P*-value < 0.05. Values for each body composition variable are displayed as mean [SD]. *P*-values for 2-way ANOVA tests to determine the effects of diet and *APOE* genotype within each sex are reported for outcomes measured after 4 months of diet at 8 months old. Sample size: *n* = 7-8/group. *APOE3*, apolipoprotein E3; *APOE4*, apolipoprotein E4; LFD, low-fat diet; HFD, high-fat diet.

#### Effects of APOE4 and HFD on Whole-Body Energy Expenditure and Substrate Use

**Figure 2.**
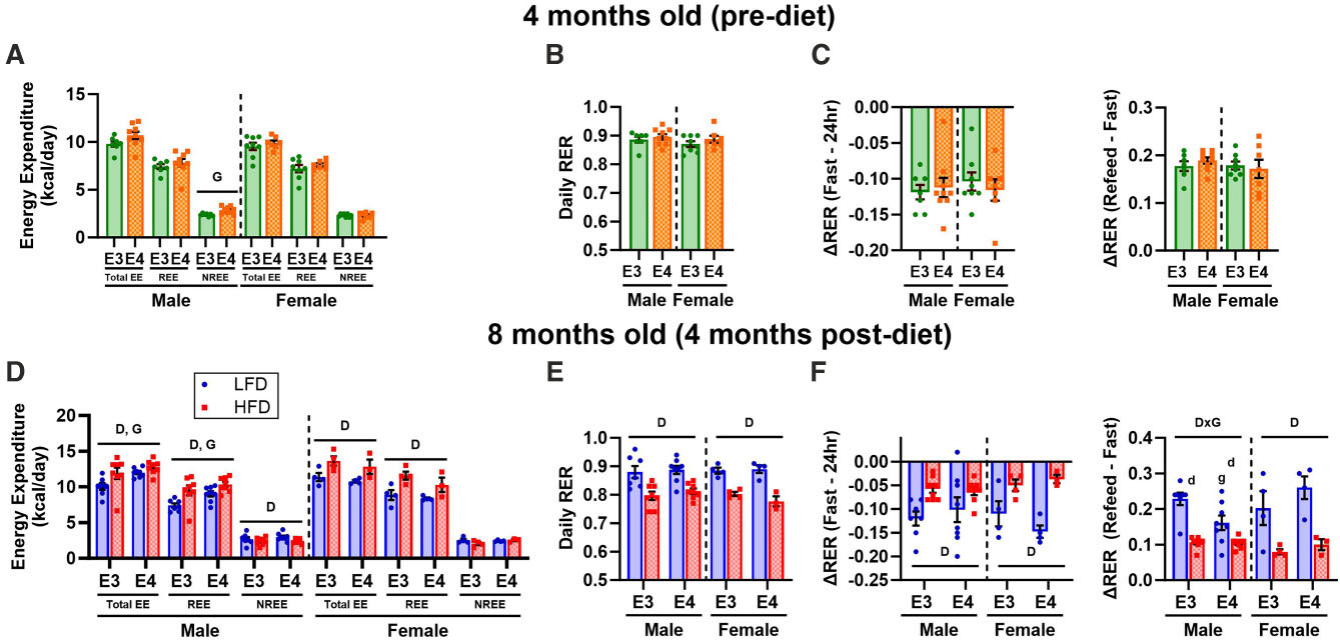
Effects of *APOE4* and HFD on unadjusted whole-body energy expenditure and respiratory exchange ratio. Indirect calorimetry data were collected prior to diet start at 4 months old and prior to sacrifice at 8 months old after 4 months of diet. Daily total (TEE), resting (REE), and non-resting (NREE) energy expenditure **(A)** and respiratory exchange ratio (RER) **(B)** at 4 months old. Change in RER during fasting and refeeding challenge at 4 months old **(C)**. Daily TEE, REE, NREE **(D)** and RER **(E)** at 8 months old. Change in RER during fasting and refeeding challenge at 8 months old **(F)**. Sample size: 4 month indirect data, *n* = 11-12/group; 8 month indirect data, *n* = 3-8/group. Values are presented as mean ± SEM. The following symbols represent comparisons with *P* ≤ 0.05: D, main effect diet; G, main effect genotype; D×G, diet by genotype interaction; d, post-hoc diet effect within genotype group; g, post-hoc genotype effect within diet group. LFD, low-fat diet; HFD, high-fat diet; E3, apolipoprotein E3; E4, apolipoprotein E4.

Since skeletal muscle is a major determinant of whole-body energy expenditure^9^ and substrate preference,^12^ we were interested in assessing these outcomes with indirect calorimetry before and after diet. Before diet, *APOE* genotype did not affect energy expenditure components in females although NREE was elevated in male *APOE4* versus *APOE3* TR mice (Figures 2A, S1A). This may be explained by the thermic effect of food since food consumption trended higher in *APOE4* TR males (*P* = 0.066) (Figure S1B). Activity levels, which also influence EE, did not differ between *APOE* genotypes in males or females (Figure S1C). *APOE* genotype did not affect daily RER or change in RER during metabolic challenge in either sex before diet (Figures 2B–C, S1D).

After 4 months of diet, HFD mice exhibited greater TEE and REE compared to LFD mice in *APOE3* and *APOE4* TR males and females (Figures 2D, S2A). In males, NREE was lower in HFD versus LFD mice which could partially result from reduced activity levels (Figures 2D, S2B). Differences in energy expenditure components between HFD and LFD mice were not significant after including fat and lean mass as co-variates, indicating these findings are driven by differences in body composition (Table 2). HFD was also associated with reduced daily RER, which is consistent with greater metabolism of lipids relatives to carbohydrates (Figures 2E, S2C). Change in RER during metabolic challenge was also lower in HFD versus LFD mice (Figure 2F).

**Table 2.** ANCOVA analysis of 8-month-old energy expenditure data co-varying for fat and lean mass.

Males											
	*APOE3*		*APOE4*		ANCOVA *P*-values			Effect of co-variate			
	LFD	HFD	LFD	HFD	Diet x genotype interaction	Diet main effect	Genotype main effect	Lean mass co-variate *P*-value	Lean mass co-variate partial eta^2^	Fat mass co-variate *P*-value	Fat mass co-variate partial eta^2^
TEE (kcal/day)	10.32 [0.563]	12.24 [0.478]	12.16 [0.392]	12.74 [0.470]	0.074	0.103	0.004*	0.020*	0.204	0.969	0.00
REE (kcal/day)	7.88 [0.598]	9.74 [0.508]	9.27 [0.416]	10.00 [0.499]	0.148	0.112	0.046*	0.568	0.014	0.377	0.033
NREE (kcal/day)	2.44 [0.240]	2.50 [0.203]	2.89 [0.167]	2.75 [0.200]	0.513	0.895	0.035*	<0.001*	0.446	0.028*	0.186
Females											
	*APOE3*		*APOE4*		ANCOVA *P*-values			Effect of co-variate			
	LFD	HFD	LFD	HFD	Diet x genotype interaction	Diet main effect	Genotype main effect	Lean mass co-variate *P*-value	Lean mass co-variate partial eta^2^	Fat mass co-variate *P*-value	Fat mass co-variate partial eta^2^
TEE (kcal/day)	13.06 [0.552]	11.62 [0.467]	12.62 [0.379]	10.78 [0.589]	0.376	0.108	0.046*	0.672	0.021	0.004*	0.620
REE (kcal/day)	9.96 [0.548]	10.02** [0.464]	9.89 [0.376]	8.86** [0.584]	0.031*	N/A	N/A	0.434	0.069	0.023*	0.452
NREE (kcal/day)	3.11 [0.446]	1.60 [0.378]	2.74 [0.306]	1.93 [0.476]	0.078	0.154	0.923	0.156	0.210	0.196	0.178

* *P*-value < 0.05

** *P*-value < 0.05 for HFD *APOE4* vs. *APOE3* comparison. Values for each energy expenditure variable are displayed as estimated marginal means [SEM]. *P*-values from ANCOVA analyses to determine the effects of diet and *APOE* genotype within each sex are reported for outcomes measured after 4 months of diet at 8 months old. Sample size: *n* = 3-8/group. TEE, total energy expenditure; REE, resting energy expenditure; NREE, non-resting energy expenditure; *APOE3*, apolipoprotein E3; *APOE4*, apolipoprotein E4; LFD, low-fat diet; HFD, high-fat diet.

*APOE* genotype also affected energy expenditure and RER after 4 months of diet. In males, *APOE4* TR mice displayed greater TEE and REE relative to *APOE3* TR mice even after co-varying for differences in fat and lean mass (Figure 2D, Table 2). After controlling for body composition, NREE was also elevated in *APOE4* versus *APOE3* TR males (Table 2) which again may reflect differences in food intake (Figure S2D). In females, *APOE* genotype did not affect unadjusted energy expenditure levels (Figure 2D). However, TEE was lower in *APOE4* versus *APOE3* TR females after co-varying for fat and lean mass and there was an interaction between diet and *APOE* genotype on REE (Table 2). Post-hoc analyses showed that *APOE4* TR females had lower REE versus *APOE3* TR females on a HFD only. Although *APOE* genotype did not impact daily RER in males or females (Figures 2E, S2C), rise in RER during refeeding was lower in LFD *APOE4* versus *APOE3* TR males, indicating reduced metabolic flexibility (Figure 2F).

#### Impact of APOE4 and HFD on Glucose Tolerance and Serum Lactate

**Figure 3.**
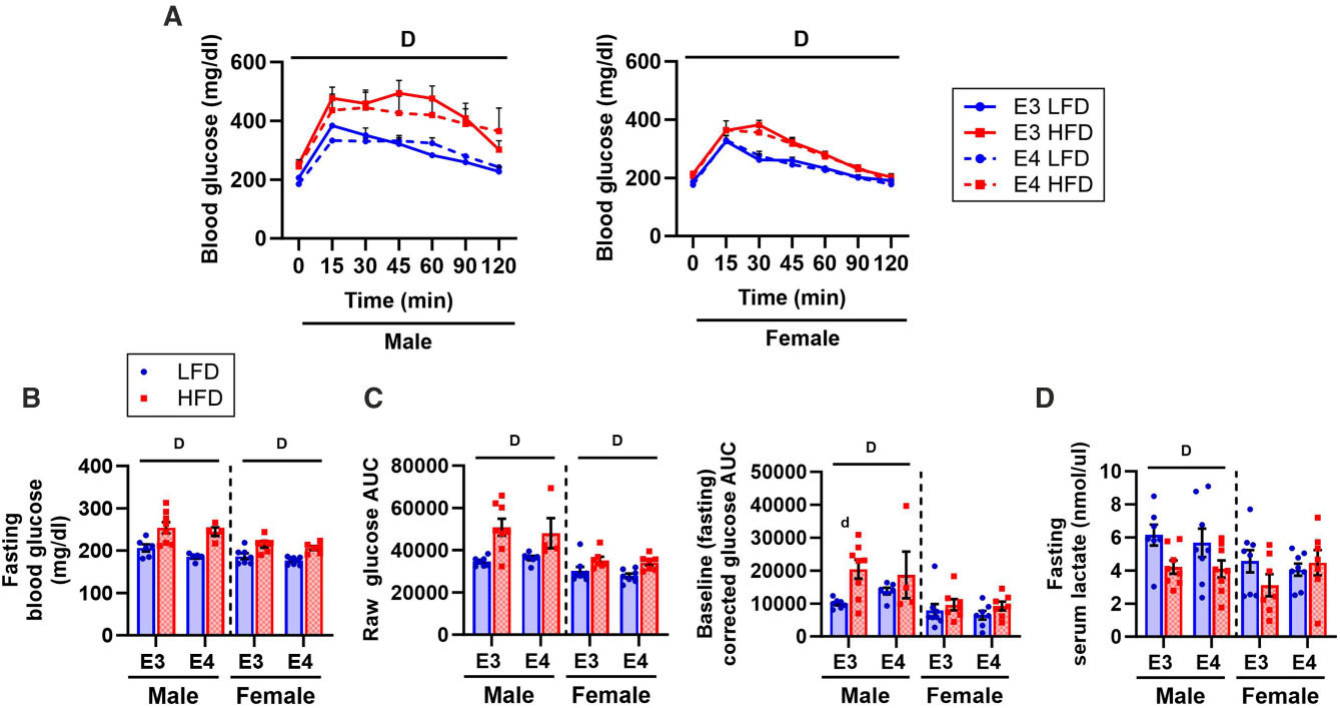
Impact of *APOE4* and HFD on glucose tolerance and serum lactate. Glucose tolerance was assessed at 7 months old after 3 months of diet and serum lactate was measured at 8 months old after 4 months of diet. Blood glucose measured during glucose tolerance testing **(A)**. There was a significant interaction between time and diet and a main effect of time (not shown). Fasting blood glucose measured prior to glucose injection **(B)**. Glucose area under the curve (AUC) calculated from raw glucose values and baseline (fasting) corrected glucose values measured during glucose tolerance test **(C)**. Fasting serum lactate levels **(D)**. Sample size: glucose tolerance, *n* = 4-8/group; serum lactate, *n* = 7-8/group. Values are presented as mean ± SEM. The following symbols represent comparisons with *P* ≤ 0.05: D, main effect diet; d, post-hoc diet effect within genotype group. LFD, low-fat diet; HFD, high-fat diet; E3, apolipoprotein E3; E4, apolipoprotein E4.

Skeletal muscle plays an important role in regulating blood glucose levels by serving as a major site of glucose disposal.^11^ We observed a significant main effect of diet on blood glucose levels in all mice during the GTT (Figure 3A). Since baseline (fasting) blood glucose levels were significantly higher in HFD versus LFD mice (Figure 3B), we calculated AUC using both raw and baseline corrected values (Figure 3C). Raw glucose AUC was greater in HFD versus LFD mice of both sexes and did not differ between *APOE3* and *APOE4* TR mice. After correcting for baseline differences in blood glucose, the effect of diet was only significant in *APOE3* TR males. We also examined fasting serum lactate since this has been proposed as a marker of metabolic health and is influenced by muscle metabolism.^33^ HFD was associated with lower serum lactate levels in males only (Figure 3D).

### Skeletal Muscle Metabolic Characterization of *APOE3* and *APOE4* TR Mice on LFD or HFD

#### Differences in Skeletal Muscle Proteome Driven By APOE4 and HFD

##### Number of Skeletal Muscle Proteins Affected By APOE4 and HFD

**Figure 4.**
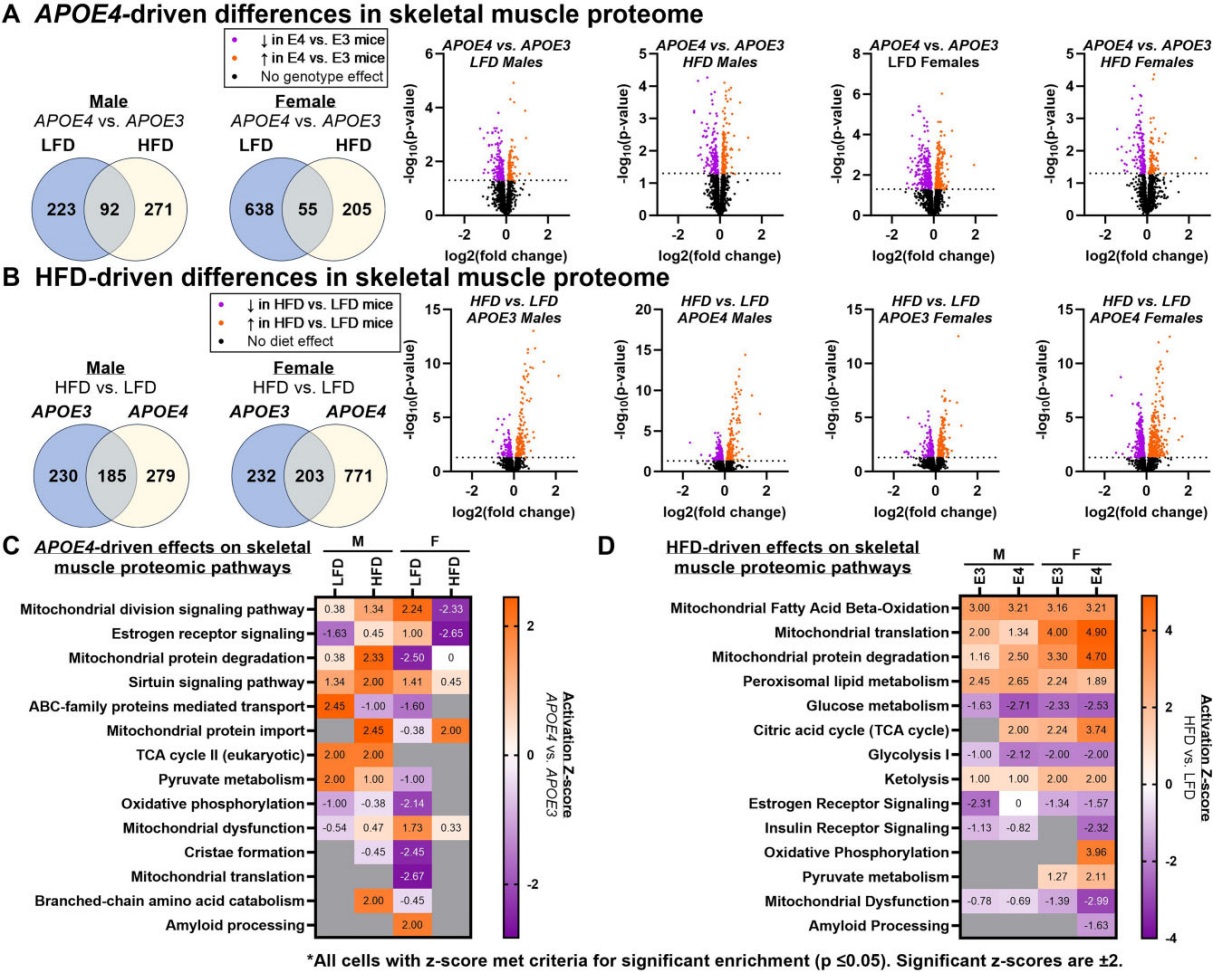
Differences in skeletal muscle proteome driven by *APOE4* and HFD. Gastrocnemius collected at sacrifice at 8 months old after 4 months of diet was used to assess the skeletal muscle proteome. Number of proteins that differed in expression in apolipoprotein E4 (*APOE4*) versus *APOE3* targeted replacement (TR) mice in males and females on a low-fat diet (LFD) or high-fat diet (HFD) displayed as Venn diagrams and volcano plots **(A)**. Number of proteins that differed in expression in HFD versus LFD mice in male and female *APOE4* and *APOE3* TR mice displayed as Venn diagrams and volcano plots **(B)**. Horizontal dotted line in volcano plot represents significance threshold (*P* ≤ 0.05) for proteins selected for further analysis. Heatmaps of pathway activation z-scores for the effect of *APOE4*  **(C)** or HFD **(D)** across groups. Activation z-scores are only shown for pathways with a significant enrichment score (*P* ≤ 0.05) and enough data to predict a z-score. Sample size: *n* = 7-8/group. M, male; F, female; ABC, ATP-binding cassette.

**Figure 5.**
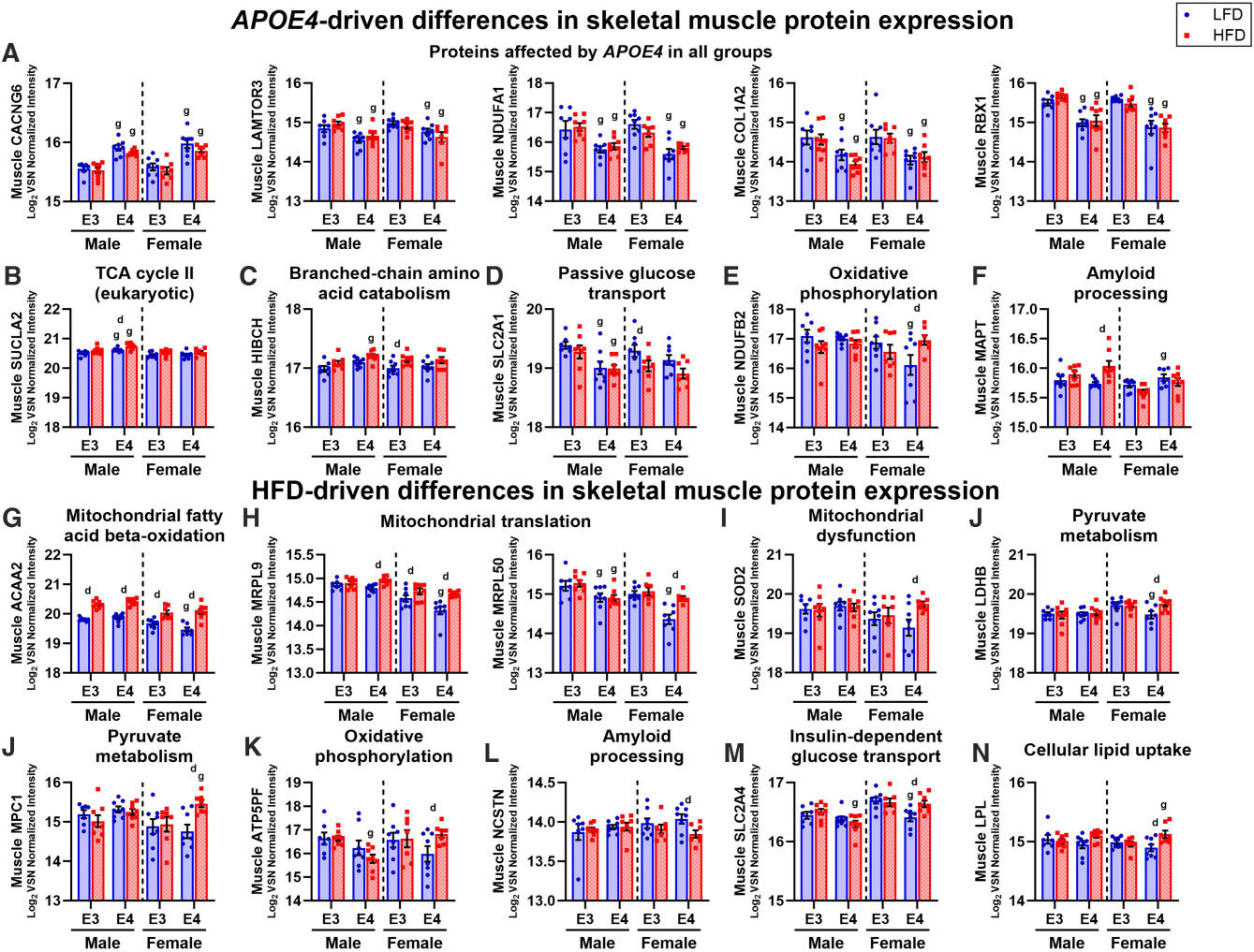
Key proteins impacted by *APOE4* and HFD in skeletal muscle. Gastrocnemius collected at sacrifice at 8 months old after 4 months of diet was used to assess the skeletal muscle proteome. Variance stabilization normalized (VSN) log_2_ transformed intensities are plotted for selected proteins for the effect of apolipoprotein E4 (*APOE4*) **(A-F)** or high-fat diet (HFD) **(G-N)**. Proteins that differed in expression between *APOE4* and *APOE3* targeted replacement (TR) mice across all groups **(A)**. Proteins representing the impact of *APOE4* on the expression of the following pathways or functions: TCA cycle II (eukaryotic) **(B)**, branched-chain amino acid metabolism **(C)**, passive glucose transport **(D)**, oxidative phosphorylation **(E)**, and amyloid processing **(F)**. Proteins representing the impact of HFD on the expression of the following pathways or functions: mitochondrial fatty acid beta-oxidation **(G)**, mitochondrial translation **(H)**, mitochondrial dysfunction **(I)**, pyruvate metabolism **(J)**, oxidative phosphorylation **(K)**, amyloid processing **(L)**, insulin-dependent glucose transport **(M)**, cellular lipid uptake **(N)**. Sample size: *n* = 7-8/group. Values are presented as mean ± SEM. The following symbols represent comparisons with *P* ≤ 0.05: d, diet effect within genotype group; g, genotype effect within diet group. LFD, low-fat diet; HFD, high-fat diet; E3, apolipoprotein E3; E4, apolipoprotein E4. Protein abbreviations: ACAA2, 3-ketoacyl-CoA thiolase, mitochondrial; ATP5PF, ATP synthase-coupling factor 6, mitochondrial; CACNG6, voltage-dependent calcium channel gamma-6 subunit; COL1A2, collagen type I alpha 2 chain; HIBCH, 3-hydroxyisobutyryl-CoA hydrolase, mitochondrial; LAMTOR3, late endosomal/lysosomal adaptor and MAPK and MTOR activator 3; LDHB, lactate dehydrogenase B; LPL, lipoprotein lipase; MAPT, microtubule-associated protein tau; MPC1, mitochondrial pyruvate carrier 1; MRPL50, large ribosomal subunit protein mL50; MRPL9, large ribosomal subunit protein bL9m; NCSTN, nicastrin (fragment); NDUFA1, NADH dehydrogenase [ubiquinone] 1 alpha subcomplex subunit 1; NDUFB2, NADH dehydrogenase [ubiquinone] 1 beta subcomplex subunit 2, mitochondrial; RBX1, ring-box 1; SLC2A1, solute carrier family 2, facilitated glucose transporter member 1; SLC2A4, solute carrier family 2, facilitated glucose transporter member 4; SOD2, superoxide dismutase, mitochondrial; SUCLA2, succinate-CoA ligase subunit beta (fragment), mitochondrial.

Variations in the skeletal muscle proteome between *APOE* genotype and diet groups in male and female mice are summarized in Figure 4. In male skeletal muscle, the expression of 315 proteins in LFD mice and 363 proteins in HFD mice differed between *APOE3* and *APOE4* TR mice (Figure 4A). Only 92 of the same proteins were altered by *APOE* genotype in LFD and HFD male mice. In female skeletal muscle, the expression of 693 proteins in LFD mice and 260 proteins in HFD mice differed between *APOE3* and *APOE4* TR mice and only 55 of these proteins were affected by *APOE* genotype on both diets. Out of all proteins that differed between *APOE4* and *APOE3* TR mice, only 5 were impacted by *APOE4* across all groups (Figure 5A). Voltage-dependent calcium channel gamma-6 subunit (CACNG6) levels were greater in *APOE4* TR mice, while late endosomal/lysosomal adaptor and MAPK and MTOR activator 3 (LAMTOR3), NADH dehydrogenase (ubiquinone) 1 alpha subcomplex subunit 1 (NDUFA1), collagen type I alpha 2 chain (COL1A2), and ring-box 1 (RBX1) levels were lower in *APOE4* TR mice. Diet comparisons showed that 415 proteins in male *APOE3* TR mice, 464 proteins in male *APOE4* TR mice, 435 proteins in female *APOE3* TR mice, and 974 proteins in female *APOE4* TR mice differed in expression between HFD and LFD groups (Figure 4B).

##### APOE4-driven Effects on Skeletal Muscle Proteomic Pathways

Pathway analysis to investigate major metabolic functions that would be affected by proteomic differences between *APOE3* and *APOE4* TR mice showed that the effects of *APOE4* largely depend on sex and diet (Figure 4C). For example, mitochondrial protein degradation was increased by *APOE4* in HFD males and reduced by *APOE4* in LFD females. Further, mitochondrial protein import was only upregulated by *APOE4* in HFD mice. Another pathway involved in metabolism, ATP binding cassette (ABC)-family mediated transport was only increased in LFD *APOE4* TR males. *APOE4* was also associated with increased pyruvate metabolism in LFD males.

Some pathways were uniquely affected by *APOE4* in males. This included TCA cycle II (eukaryotic), which was upregulated in *APOE4* versus *APOE3* TR males on both diets, as reflected by the expression of succinate-CoA ligase subunit beta (SUCLA2) (Figure 5B). Branched-chain amino acid catabolism was only increased in HFD *APOE4* versus *APOE3* TR males. 3-hydroxyisobutyryl-CoA hydrolase (HIBCH), an enzyme involved in the breakdown of valine, represents one of the proteins involved in branched-chain amino acid catabolism that drove this finding (Figure 5C). We also searched our dataset for select proteins implicated in nutrient flux and found that facilitated glucose transporter member 1 (SLC2A1), which mediates basal glucose uptake, is lower in *APOE4* versus *APOE3* TR males on both diets (Figure 5D).

Other pathways were uniquely affected by *APOE4* in females. Mitochondrial division signaling was increased by *APOE4* in LFD females yet decreased by *APOE4* in HFD females. For several pathways, the effect of *APOE4* was unique to LFD females. This included oxidative phosphorylation, cristae formation, and mitochondrial translation, which were all significantly reduced in LFD *APOE4* versus *APOE3* TR females. NADH dehydrogenase 1 beta subcomplex subunit 2 (NDUFB2), an electron transport chain (ETC) complex I subunit, represents one of the proteins in the oxidative phosphorylation pathway that was decreased in LFD *APOE4* versus *APOE3* TR females (Figure 5E). Amyloid processing was also only affected in LFD females and was significantly increased by *APOE4*, which was partially driven by elevated levels of microtubule-associated protein tau (MAPT) (Figure 5F). This protein becomes hyperphosphorylated and aggregates in the ad brain, a process that may be facilitated by amyloid beta (Aβ), which also clumps together in the ad brain and is a byproduct of amyloid processing.^34^

Although our results indicate that *APOE4*-associated differences in pathway expression generally depend on sex and diet, sirtuin signaling was significantly enriched (*P* ≤ 0.05) in all groups and the activation z-score was positive for all comparisons between *APOE4* and *APOE3* TR mice (Figure 4C). However, the z-score only reached significance in HFD males. Interestingly, sirtuin signaling was least affected by *APOE4* in HFD females (z = 0.45). The proteins that drove this finding were highly variable between groups (Figure S3A).

##### Diet-driven Effects on Skeletal Muscle Proteomic Pathways

Unlike the effects of *APOE4* which varied substantially across sex and diet groups, HFD altered the expression of several pathways in the same direction based on trending or significant activation z-scores across all groups (Figure 4D). This included mitochondrial fatty acid β-oxidation, mitochondrial translation, mitochondrial protein degradation, peroxisomal lipid metabolism, and ketolysis, which were all increased in HFD versus LFD mice. Other pathways such as glucose metabolism, glycolysis, and mitochondrial dysfunction were reduced by HFD in all groups. For pathways with similar z-score values across groups, such as mitochondrial fatty acid β-oxidation, many of the same proteins were affected by HFD in all groups as shown by increased 3-ketoacyl-CoA thiolase (ACAA2) in HFD versus LFD mice (Figure 5G). In other cases where absolute z-score values were higher in *APOE4* TR females such as for mitochondrial translation, mitochondrial protein degradation, and mitochondrial dysfunction, more proteins were altered by HFD in *APOE4* TR females compared to other groups. Proteins that drove these findings include MRPL9 and MPRL50 for mitochondrial translation (Figure 5H) and superoxide dismutase (SOD2) for mitochondrial dysfunction (Figure 5I).

Interestingly, the effect of diet on the direction of pathway expression was not universal across sex and *APOE* groups. For example, the citric acid cycle was increased by HFD in females of both *APOE* genotypes but only *APOE4* TR males. Insulin receptor signaling was significantly reduced in *APOE4* TR females and trended lower in males. Two pathways downstream of insulin receptor signaling, pyruvate metabolism and oxidative phosphorylation, were only mainly affected in female *APOE4* TR mice. Pyruvate metabolism was greater in HFD versus LFD *APOE4* TR females, due to increased levels of proteins like lactate dehydrogenase B (LDHB) and mitochondrial pyruvate carrier 1 (MPC1) (Figure 5J). Pyruvate metabolism trended in the same direction in *APOE3* TR females. Oxidative phosphorylation was only elevated in HFD versus LFD *APOE4* TR females, as represented by levels of ATP synthase-coupling factor 6 (ATP5PF) (Figure 5K) and NDUFB2 (Figure 5E). The expression of 26 proteins involved in oxidative phosphorylation were altered by HFD in *APOE4* TR females compared to only 3 proteins in other groups (Figure S3B). Among the 26 oxidative phosphorylation proteins altered by HFD in *APOE4* TR females, all ETC complexes are represented. Notably though, while most of these proteins were increased by HFD, mitochondrial DNA-encoded proteins ATP synthase subunit a (MT-ATP6) and NADH-ubiquinone oxidoreductase chain 3 (MT-ND3) were both reduced in HFD versus LFD *APOE4* TR females. These differences did not appear to be related to differences in mitochondrial protein abundance (Figure S3C). Interestingly, HFD was also associated with a trend towards downregulated amyloid processing in *APOE4* TR females. This is reflected in part by decreased levels of nicastrin (NCSTN) in HFD versus LFD *APOE4* TR females (Figure 5L), which is part of the gamma-secretase complex that cleaves amyloid precursor protein and is necessary for beta-secretase-driven production of Aβ.^35^ In addition to investigating proteins that drove pathway analysis results, we further assessed specific proteins that are central to regulating nutrient flux in skeletal muscle. Of note, we found that facilitated glucose transporter member 4 (SLC2A4), which mediates insulin-dependent glucose uptake into skeletal muscle, was lower in LFD *APOE4* versus *APOE3* TR females and higher in HFD versus LFD *APOE4* TR females (Figure 5M). This same transporter was reduced by *APOE4* in HFD males. We also found that the lipoprotein lipase (LPL) expression was greater in HFD *APOE4* versus *APOE3* TR females and in HFD versus LFD *APOE4* TR females (Figure 5N).

##### Opposing Effects of APOE4 and HFD on Skeletal Muscle Proteome in Female Subgroups

Given that *APOE4* had the greatest effect on protein expression in LFD females and HFD had the greatest effect on protein expression in *APOE4* females, we were interested in comparing these outcomes. Out of the 693 proteins that differed between LFD *APOE4* and *APOE3* TR females and the 974 proteins that differed between HFD and LFD *APOE4* TR females, 329 of these proteins were the same (Figure S3D). Comparison analysis showed that several metabolic pathways were regulated in the opposite direction (Figure S3E). Mitochondrial translation, mitochondrial protein degradation, oxidative phosphorylation, and cristae formation were reduced in *APOE4* versus *APOE3* TR LFD females yet increased in *APOE4* TR females on a HFD versus LFD based on trending or significant activation z-scores. Mitochondrial dysfunction showed the opposite relationship. Of the proteins involved in oxidative phosphorylation affected in the reverse direction between these groups, the majority are ETC complex V subunits (Figure S3F).

#### Influence of APOE Genotype and Diet on Skeletal Muscle Mitochondrial Function

**Figure 6.**
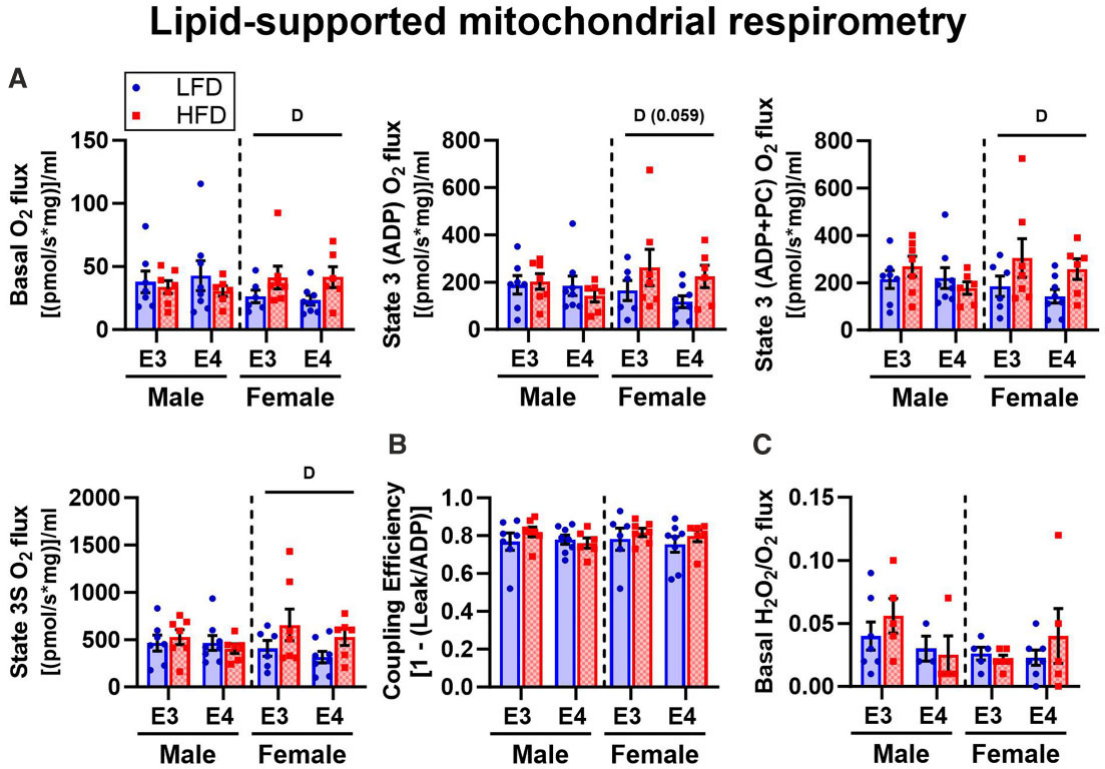
Influence of *APOE4* and HFD on lipid-supported mitochondrial respiration in skeletal muscle. Quadriceps collected at sacrifice at 8 months old after 4 months of diet was used to assess respiration in isolated mitochondria. Lipid-stimulated mitochondrial oxygen consumption measured under basal (2 m m malate, 0.01 m m CoA, 2.5 m m carnitine, and 0.01 m m palmitoyl-CoA), state 3 (2.5 m m ADP), state 3 + PC (0.01 m m palmitoyl-carnitine, PC), and state 3S (10 m m succinate) conditions **(A)**. Coupling efficiency calculated from leak (basal) and state 3 respiration **(B)**. H_2_O_2_/O_2_ flux ratio calculated from basal H_2_O_2_ and O_2_ flux **(C)**. Sample size: respirometry data, *n* = 6-8/group; H_2_O_2_ data, *n* = 3-7/group. Values are presented as mean ± SEM. The following symbols represent comparisons with *P* ≤ 0.05: D, main effect diet. Symbols with *P*-values in parenthesis indicate trending effects. LFD, low-fat diet; HFD, high-fat diet; E3, apolipoprotein E3; E4, apolipoprotein E4.

We wanted to know if *APOE4*- and HFD-driven effects on mitochondrial proteomic pathways in skeletal muscle translated to functional outcomes. We first did this by assessing lipid- and carbohydrate-supported respiration in isolated skeletal muscle mitochondria under saturating ADP conditions. Lipid-supported mitochondrial O_2_ flux was not affected by *APOE* genotype, sex, or diet in males. However, O_2_ flux was greater in female HFD versus LFD mice under lipid-supported conditions regardless of *APOE* genotype (Figure 6A), which is consistent with HFD-driven elevations in skeletal expression of pathways related to lipid metabolism in this group. This was significant during basal, state 3 + PC, and state 3S respiration, and trending for state 3 (ADP) respiration (*P* = 0.059). Coupling efficiency, which reflects the degree of linkage between electron transport and ATP production, and H_2_O_2_/O_2_ flux ratio were not affected by *APOE4* or HFD (Figure 6B–C). Under carbohydrate-supported conditions, mean O_2_ flux was generally higher in HFD versus LFD *APOE4* TR females, but this effect was not significant (Figure S4A). Coupling efficiency and H_2_O_2_/O_2_ flux ratio did not differ between groups (Figure S4B, C). We also assessed mitochondrial response to changes in energetic demand under carbohydrate-supported conditions using a modified CK clamp assay (Figure S4D).^24^ There were no significant differences in mitochondrial conductance, calculated from the slope of the relationship between ∆G_ATP_ and O_2_ flux (Figure S4E).^25^

#### Role of APOE Genotype and Diet in Fiber-Type Distribution and Lipid Droplet Accumulation in Skeletal Muscle

**Figure 7.**
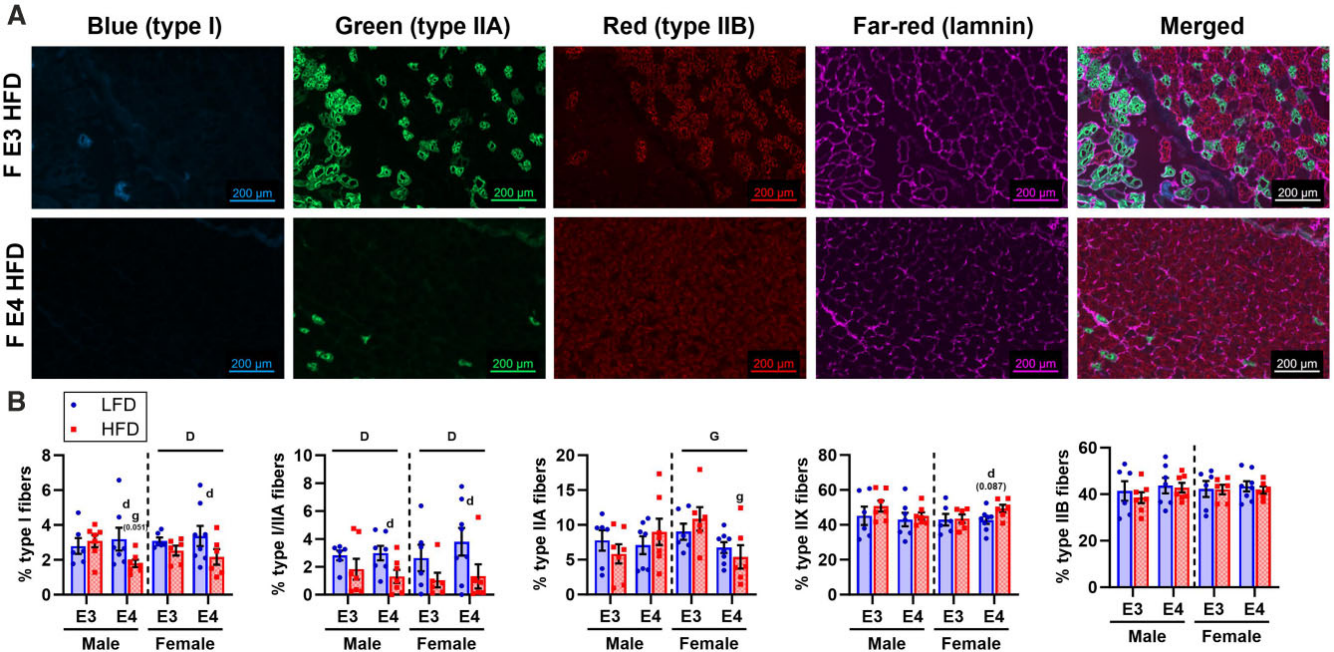
Impact of *APOE4* and HFD on fiber-type distribution in skeletal muscle. Quadriceps collected at sacrifice at 8 months old after 4 months of diet was used to assess fiber-type distribution by staining for the muscle fiber membrane (laminin) and fiber types I, IIA, and IIB (unstained fibers represent type IIX fibers). Single-channel images are shown for select mice to represent *APOE* genotype effect on percentage of type IIA (green) fibers in high-fat diet females **(A)**. Percentage of type I, I/IIA, IIA, IIB, and IIX fibers in whole-tissue sections was determined using MuscleJ2 **(B)**. Sample size: *n* = 6-8/group. Values are presented as mean ± SEM. The following symbols represent comparisons with *P* ≤ 0.05: D, main effect diet; G, main effect genotype; d, post-hoc diet effect within genotype group; g, post-hoc genotype effect within diet group. Symbols with p-values in parenthesis indicate trending effects. F, female; LFD, low-fat diet; HFD, high-fat diet; E3, apolipoprotein E3; E4, apolipoprotein E4.

Since mitochondrial metabolism in skeletal muscle varies by fiber-type and is influenced by fuel storage, we also assessed fiber-type distribution and lipid droplet accumulation in quadricep muscle sections. On average, approximately 3447 fibers were analyzed for fiber-type per mouse using MuscleJ2. Fiber-type distribution was dependent on both diet and *APOE* genotype (Figure 7, S5). HFD was associated with a shift away from oxidative fibers in *APOE4* TR mice, as represented by a lower percentage of type I (slow oxidative) and I/IIA hybrid fibers in male and female *APOE4* versus *APOE3* TR mice. *APOE4* was linked to less type IIA fibers (fast oxidative/glycolytic) but only in HFD females, as reflected by the lower percentage of type IIA fibers in HFD *APOE4* versus *APOE3* TR females. Staining for lipid droplets with BODIPY 493/503 showed visual accumulation of lipid droplets in HFD but not LFD mice regardless of *APOE* genotype or sex and lipid droplets were primarily located in type IIA fibers (Figure S6).

## Discussion

While skeletal muscle contributes substantially to whole-body metabolism,^9,11,12^ which is tied to ad risk through genetic and lifestyle factors,^4,5,36,37^ the potential role of skeletal muscle in ad progression is unclear. Here, we provided novel evidence that the strongest genetic risk factor for ad, *APOE4*, is associated with HFD-dependent reductions in energy expenditure in female mice and alterations in skeletal muscle mitochondrial pathways in male and female mice. Processes that support mitochondrial energy metabolism are generally upregulated in males, downregulated in females, and modified by diet. These differences are observed at a time that would predate ad development in an age-equivalent human, highlighting the early role diet may play in *APOE4*-mediated disease.

*APOE4* has been linked to earlier and faster reductions in weight and BMI and lower fat mass during early stages of cognitive decline, particularly in women.^38–42^ However, the potential interacting role of diet and corresponding effects on whole-body metabolism have not been thoroughly examined. Our assessment showed that *APOE4* and HFD act independently to influence body anthropometrics. HFD had the expected effects of increasing body and fat mass while reducing % lean mass. *APOE4* was associated with lower % lean mass in males and a trend towards lower absolute lean mass in females at 8 months old. While this agrees with human studies reporting reduced lean mass in early stages of ad,^16^ the impact of *APOE4* on lean mass in humans is less clear. In *APOE4* versus *APOE3* TR males, we also observed greater gains in fat mass and lower gains in lean mass. This contrasts human studies reporting lower fat mass in women *APOE4* carriers versus *APOE4* non-carriers during early ad stages, without a genotype effect in males.^38^ These differences could be explained by species-specific effects of *APOE4* or a complex interaction between several factors, including *APOE4*, age, sex, and diagnosis on body composition that are not captured in our study.

To evaluate aspects of whole-body metabolism tied to skeletal muscle function, we examined energy expenditure,^9^ substrate preference (RER),^12^ and glucose tolerance,^11^ Starting with energy expenditure, we found that 8-month-old *APOE4* versus *APOE3* TR males have greater TEE, REE, and NREE after controlling for body composition. While feeding behavior likely influenced NREE, our finding that *APOE4* also affects REE suggests *APOE4* impacts energy expenditure through mechanisms separate from feeding- and activity-related behavior. Additional studies are needed to determine what is responsible for elevated REE in *APOE4* TR males. *APOE4*-driven elevations in REE in the setting of increased food consumption and weight gain may reflect imbalances in energy homeostasis. This could be mediated by skeletal muscle factors not explored in this study, such as differences in in vivo metabolic demands not accounted for in isolated mitochondria, or by metabolic activity in other tissues (ie, adipose tissue, liver, brain). In females, we observed the opposite effect of *APOE4* on energy expenditure at 8 months old. After adjusting for body composition, female *APOE4* versus *APOE3* TR mice had lower TEE on both diets and lower REE on a HFD only. This is consistent with human studies demonstrating lower REE in young female *APOE4* carriers versus non-carriers^20^ and suggests that a diet high in saturated fats may influence this relationship. Although we found that type IIA fibers, which are mitochondrial-rich relative to other type II fiber subtypes,^43^ are reduced in HFD *APOE4* versus *APOE3* TR females, we did not observe differences in mitochondrial protein abundance or mitochondrial respiration. More work is needed to determine what factors and/or other tissues mediate these findings.

We also observed that the impact of *APOE4* on metabolic flexibility and glucose tolerance is diet-specific and limited to males. While *APOE4* did not affect substrate preference as reflected by daily RER, LFD *APOE4* TR males exhibited a blunted ability to increase RER during feeding after a prolonged fast compared to LFD *APOE3* TR males. This suggests that *APOE4* may impair the ability to switch from lipid to carbohydrate metabolism in LFD males, a sign of metabolic inflexibility. As expected, HFD was also associated with worse metabolic flexibility, which could explain the lack of *APOE* genotype effect on change in RER during metabolic challenge in HFD males. A similar reasoning may account for our observation that HFD was linked to decreased glucose tolerance, determined by greater baseline corrected glucose AUC values, in *APOE3* TR males, but not *APOE4* TR males. That said, this interpretation is limited by the lack of *APOE* genotype effect on glucose AUC. Even though *APOE* genotype did not affect glucose tolerance, *APOE4* TR males had lower skeletal muscle levels of SCLC2A1 (GLUT1) on both diets and SLC2A4 (GLUT4) on a HFD, which mediate insulin-independent and insulin-dependent glucose uptake into skeletal muscle respectively and this same trend was observed for SLC2A4 in LFD females.

Although we did not detect differences in skeletal muscle mitochondrial respiratory function to explain the effects of *APOE4* on whole-body metabolism, our proteomic analyses showed that *APOE4* changes the expression of several metabolic pathways in skeletal muscle in a way that largely varies between diet conditions. For example, ABC-family mediated transport, which uses ATP to move molecules such as lipids and Aβ across cell membranes and has been implicated in ad pathogenesis,^44^ was upregulated by *APOE4* in LFD males only. Branched-chain amino acid catabolism, which may also be involved in AD,^45^ along with mitochondrial protein degradation and mitochondrial protein import were also increased by *APOE4*, but only in HFD males. On the other hand, TCA cycle pathway expression was upregulated by *APOE4* in males on both diets. Since there were no corresponding *APOE4*-associated changes in skeletal muscle mitochondrial respiration, this could reflect an adaptive mechanism to sustain energy needs.

In females, we found that several of the same skeletal muscle proteomic pathways affected by *APOE4* were also impacted by HFD. This included oxidative phosphorylation and cristae formation, which were reduced by *APOE4* in LFD mice. These same mitochondrial pathways were unaffected by *APOE4* in HFD mice and increased by HFD in *APOE4* TR females. In neuronal cell models, *APOE4* has been linked to reduced expression of all ETC complexes^46^ and our results indicate this may also occur in skeletal muscle, which could be detrimental to mitochondrial function over time. Alternatively, reduced oxidative phosphorylation proteins in *APOE4* versus *APOE3* TR females on a LFD, without an *APOE* genotype effect in HFD females or differences in mitochondrial respiration, could signify greater mitochondrial efficiency. On a HFD, the opposite may be true. Greater levels of oxidative phosphorylation proteins on a HFD versus LFD in *APOE4* TR females only, with similar diet effects on mitochondrial respiration regardless of *APOE* genotype, could reflect an effort to maintain normal mitochondrial energy metabolism though protein upregulation. This is consistent with findings that genes involved in mitochondrial energy production are upregulated in areas of the brain most impacted by ad pathology and oxidative stress in a familial ad mouse model.^47^ While *APOE4*-mediated effects on skeletal muscle mitochondrial pathways did not translate to differences in mitochondrial function, HFD-driven elevations in pathways supporting lipid metabolism occurred with greater lipid-supported mitochondrial respiration in females. This congruency between molecular and functional outcomes did not hold true for carbohydrate metabolism. Pyruvate metabolism was uniquely elevated by HFD in *APOE4* female muscle, which was driven by proteins like MPC1, 1 of 2 MPC subunits that serve as an essential entry point for pyruvate into mitochondria.^48^ and LDH-B, which favors the conversion of lactate into pyruvate. Despite increased expression of this pathway, along with an upregulation in oxidative phosphorylation, carbohydrate/pyruvate-supported mitochondrial respiration was not significantly affected by HFD in *APOE4* TR females. HFD-associated alterations in mitochondrial pathways at the protein level could therefore represent a compensatory response to maintain skeletal muscle bioenergetic function.

Although *APOE4*-dependent differences in the skeletal muscle proteome largely varied between diet and sex groups, some findings were common across groups. Sirtuin signaling promotes metabolic adaptations under conditions of high energetic demand^49^ and was significantly affected based on enrichment *P*-values in all mice regardless of sex. Further, the z-score for this pathway was positive for all *APOE4* versus *APOE3* comparisons, although this was only significant in HFD males. We have shown that sirtuin signaling is downregulated by *APOE4* in female muscle at 4 months old.^19^ suggesting a potential interaction between *APOE* genotype and age on this pathway. The expression of CACNG6, NDUFA1, LAMTOR3, COL1A2, and RBX1 were also altered by *APOE4* across all groups. CACNG6, which is involved in limiting cellular influx of calcium,^50^ was increased in *APOE4* TR mice. This is consistent with our previous findings that CACNG6 is elevated in skeletal muscle of 4 month old *APOE4* TR mice.^19^ We further found that NDUFA1, a subunit of mitochondrial ETC complex I, LAMTOR3, a protein involved in amino acid sensing, COL1A2, an important structural component of the extracellular matrix,^51^ and RBX1, which is involved in protein ubiquitination and turnover, were all reduced in *APOE4* versus *APOE3* muscle. Additional studies are needed to determine if these proteins are also affected in humans and can be linked to clinically relevant outcomes.

An unanswered question remains–what are the ramifications of skeletal muscle mitochondrial effects of *APOE4* on the brain and how might diet and sex influence this relationship? Skeletal muscle mitochondrial respiration is reduced in humans during early cognitive decline and is a major determinant of cardiorespiratory fitness, which is decreased in ad and positively correlated with brain volume.^10,18,52^ While unaffected by *APOE4* in our study, functional mitochondrial changes associated with differences in skeletal muscle proteomic pathways that are directly involved in energy production (eg, TCA cycle, branched-chain amino acid catabolism, mitochondrial protein import, oxidative phosphorylation, cristae formation) at later ages and in humans cannot be ruled out. This relationship is important to investigate, particularly if skeletal muscle mitochondria influence or reflect brain health and can be modified with diet. One potential mechanism linking skeletal muscle and brain outcomes is through the secretion of neuroprotective myokines during exercise. One of these myokines, irisin, is reduced in AD brain tissue and increases in plasma as exercise intensity and metabolic demand ramps up.^53,54^ Thus, irisin secretion and other myokines may be limited by skeletal muscle mitochondrial oxidative capacity. While muscle mitochondria may contribute to ad pathology through myokine section or alterative mechanisms, it's also possible that *APOE4*-mediated alterations in skeletal muscle do not affect the brain. Even so, if *APOE4* induces similar mitochondrial changes in skeletal muscle and brain tissue, this raises the possibility of using skeletal muscle as a preclinical ad biomarker in *APOE4* carriers.

Our findings are supported by the thorough assessment of skeletal muscle and whole-body metabolic outcomes and the use of multiple techniques to assess mitochondrial respiration. Due to limited tissue, gastrocnemius was used for proteomics while quadriceps was used for respirometry and tissue staining. However, both are mixed fiber-type muscles, supporting the selection of these 2 muscles. We are limited in our ability to draw conclusions about sex-dependent differences since we did not directly compare males and females, which was outside the scope of this current study. We also did not account for estrous cycle. However, C57BL/6 mice generally stop cycling between the ages of 11 and 16 months^55^ so it is likely that our mice, sacrificed at 8 months old, were cycling throughout the entirety of our study. Additional studies are needed to assess the potential interaction between sex, diet, and *APOE* genotype. Finally, we are limited by our model. Mice require genetic manipulation to induce ad neuropathology to a similar degree as humans and differences in lipid metabolism between species may complicate translational interpretation of our findings.

In summary, our work in an ad risk mouse model indicates that *APOE4* drives diet-dependent reductions in energy expenditure in females and alterations in skeletal muscle mitochondrial pathways in both sexes, without affecting mitochondrial respiratory function. Additional studies are needed to understand the long-term, functional consequences of *APOE4*-mediated, diet-dependent effects on the skeletal muscle proteome (eg, elevated branched-chain amino acid catabolism in HFD males, lower oxidative phosphorylation in LFD females). Overall, our findings support further investigation into the role of early life dietary habits in modulating skeletal muscle metabolism and ad susceptibility in *APOE4* carriers.

## Supplementary Material

zqaf017_Supplemental_Files

## Data Availability

The dataset used in this study has been uploaded to the Harvard Dataverse repository (10.7910/DVN/GR4T84).
