# Supplementary material for: APOE4 Exerts Partial Diet-dependent Effects on Energy Expenditure and Skeletal Muscle Mitochondrial Pathways in a Preclinical Model
Source: Function (Oxf). 2025 Mar 25;6(2):zqaf017. doi: 10.1093/function/zqaf017 (PMC11980864; doi:10.1093/function/zqaf017)
Supplement: zqaf017_Supplemental_Files [file zqaf017_supplemental_files.zip › Supplemental tables and figure legends.docx]

**Supplemental table 1. Influence of *APOE4* on body composition before diet start.** Values for each body composition variable are displayed as mean [SD]. P-values for t-tests to determine the effect of *APOE* genotype within each sex are reported for outcomes measured before diet start at 4 months old. Sample size: n=7-8/group. *APOE3*, apolipoprotein E3; *APOE4*, apolipoprotein E4.

| Males | | | |
| --- | --- | --- | --- |
|  | *APOE3* | *APOE4* | p-value |
| Body mass (g) | 28.2 [2.11] | 29.5 [2.08] | 0.089 |
| Fat mass (g) | 3.47 [1.15] | 3.61 [1.00] | 0.721 |
| Fat mass (%) | 12.14 [3.34] | 12.20 [3.07] | 0.957 |
| Lean mass (g) | 22.89 [1.48] | 24.01 [1.78] | 0.070 |
| Lean mass (%) | 81.29 [3.44] | 81.33 [3.10] | 0.975 |
| Females | | | |
|  | *APOE3* | *APOE4* | p-value |
| Body mass (g) | 24.4 [2.15] | 23.2 [1.47] | 0.085 |
| Fat mass (g) | 3.25 [0.79] | 2.79 [0.77] | 0.120 |
| Fat mass (%) | 13.17 [2.40] | 11.99 [3.00] | 0.244 |
| Lean mass (g) | 19.16 [1.58] | 18.61 [1.30] | 0.311 |
| Lean mass (%) | 78.50 [2.62] | 80.14 [3.35] | 0.146 |

**Supplemental figure 1. Supplemental 4-month indirect calorimetry data.** Total (TEE), resting (REE), and non-resting (NREE) energy expenditure during dark and lights cycles not adjusted for differences in body composition **(A)**. Food consumed prior to fasting and during refeed **(B)**. Daily distance traveled in cages reported **(C)**. Respiratory exchange ratio (RER) during dark and light cycles **(D)**. Sample size: n=11-12/group. Values are presented as mean ± SEM. The following symbols represent comparisons with p ≤0.05: G, genotype effect. Symbols with p-values in parenthesis indicate trending effects. E3, apolipoprotein E3; E4, apolipoprotein E4.

**Supplemental figure 2. Supplemental 8-month indirect calorimetry data.** Total (TEE), resting (REE), and non-resting (NREE) energy expenditure during dark and light cycles not adjusted for differences in body composition **(A)**. Daily distance traveled in cages **(B)**. Respiratory exchange ratio (RER) during dark and light cycles **(C)**. Food consumed prior to fasting and during refeed **(D)**. Sample size: n= 3-8/group. Values are presented as mean ± SEM. The following symbols represent comparisons with p ≤0.05: D, main effect diet; G, main effect genotype. Symbols with p-values in parenthesis indicate trending effects. LFD, low-fat diet; HFD, high-fat diet; E3, apolipoprotein E3; E4, apolipoprotein E4.

**Supplemental figure 3. Supplemental proteomic data.** Heatmap representing the log_2_(fold change) expression of all proteins involved in sirtuin signaling that significantly differed in apolipoprotein E4 (*APOE4*) versus *APOE3* targeted replacement (TR) mice for at least one comparison **(A)**. Heatmap representing the log_2_(fold change) expression of all proteins involved in the oxidative phosphorylation pathway that significantly differed in high-fat diet (HFD) versus low-fat diet (LFD) mice for at least one comparison **(B)**. Proteins are grouped by their association with complexes I-V of the electron transport chain. Grey cells in A and B represent proteins that did not statistically differ between groups. Mitochondrial protein abundance determined by percentage of summed mitochondrial protein intensity out of total summed protein intensity **(C)**. Overlap in the number of proteins that were significantly altered by *APOE4* in LFD female mice and by HFD in female *APOE4* TR mice **(D).** Heat map of activation z-scores comparing the effect of *APOE* genotype (*APOE4* versus *APOE3*) in LFD female mice compared to the effect of diet (HFD versus LFD) in *APOE4* female mice based on analysis of all proteins altered in each group. All pathways shown were significantly enriched (p ≤0.05) **(E)**. Heat map representing the log_2_(fold change) expression of all proteins in the oxidative phosphorylation pathway that significantly differed in the opposite direction for the effect of *APOE4* in LFD females and the effect of HFD in *APOE4* TR females **(F)**. Sample size: n=7-8/group. M, male; F, female. Protein abbreviations: ACADL, long-chain specific acyl-CoA dehydrogenase, mitochondrial; ACLY, ATP-citrate synthase; ACSS2, propionate-CoA ligase; ATG3, ubiquitin-like-conjugating enzyme ATG3; ATP5F1A, ATP synthase subunit alpha, mitochondrial; ATP5F1B, ATP synthase subunit beta, mitochondrial; ATP5F1C, ATP synthase subunit gamma; ATP5F1D, ATP synthase F1 subunit delta; ATP5F1E, ATP synthase subunit epsilon, mitochondrial; ATP5ME, ATP synthase subunit e, mitochondrial; ATP5MG, ATP synthase subunit g, mitochondrial; ATP5PB, ATP synthase subunit b; ATP5PD, ATP synthase subunit d, mitochondrial; ATP5PF, ATP synthase-coupling factor 6, mitochondrial; ATP5PO, ATP synthase subunit O, mitochondrial; ATPAF1, ATP synthase mitochondrial F1 complex assembly factor 1; ATPAF2, ATP synthase mitochondrial F1 complex assembly factor 2; BAX, BCL2 associated-X protein (Fragment); COX5A, cytochrome c oxidase subunit 5A, mitochondrial; CPT1B, carnitine O-palmitoyltransferase 1, muscle isoform; CYC1, cytochrome c1, heme protein, mitochondrial; CYCS, cytochrome c, somatic; Cyct, cytochrome c, testis-specific; CYGB, cytoglobin; DMAC2L, ATP synthase subunit s, mitochondrial; G6PD, glucose-6-phosphate 1-dehydrogenase; gLUD1, glutamate dehydrogenase 1, mitochondrial; H1f3, histone H1.3; LDHA, L-lactate dehydrogenase A chain; LDHB, L-lactate dehydrogenase B chain; LDHD, probable D-lactate dehydrogenase, mitochondrial; MAP1LC3A, microtubule-associated proteins 1A/1B light chain 3A; MAP1LC3B, microtubule-associated protein 1 light chain 3 beta; MAPK12, mitogen-activated protein kinase 12; MT-ATP6, ATP synthase subunit a; MT-ND3, NADH-ubiquinone oxidoreductase chain 3; NAMPT, nicotinamide phosphoribosyltransferase; NCSTN, nicastrin; NDUFA1, NADH dehydrogenase [ubiquinone] 1 alpha subcomplex subunit 1; NDUFA11, NADH dehydrogenase [ubiquinone] 1 alpha subcomplex subunit 11; NDUFA4, cytochrome c oxidase subunit NDUFA4; NDUFA7, NADH dehydrogenase [ubiquinone] 1 alpha subcomplex subunit 7; NDUFB11, NADH dehydrogenase [ubiquinone] 1 beta subcomplex subunit 11, mitochondrial; NDUFB2, NADH dehydrogenase [ubiquinone] 1 beta subcomplex subunit 2, mitochondrial; NDUFS6, NADH dehydrogenase [ubiquinone] iron-sulfur protein 6, mitochondrial; NDUFV3, NADH dehydrogenase [ubiquinone] flavoprotein 3, mitochondrial; PGAM1, phosphoglycerate mutase; PGAM2, phosphoglycerate mutase 2; PGK1, phosphoglycerate kinase 1; PRKAA2, 5'-AMP-activated protein kinase catalytic subunit alpha-2; SDHA, succinate dehydrogenase [ubiquinone] flavoprotein subunit, mitochondrial; SDHB, succinate dehydrogenase [ubiquinone] iron-sulfur subunit, mitochondrial (Fragment); SDHC, succinate dehydrogenase cytochrome b560 subunit, mitochondrial; SIRT3, NAD-dependent protein deacetylase sirtuin-3; SLC2A1, solute carrier family 2, facilitated glucose transporter member 1; SURF1, SURF1-like protein (Fragment); TIMM22, translocase of inner mitochondrial membrane 22; TIMM44, mitochondrial import inner membrane translocase subunit TIM44; TIMM50, mitochondrial import inner membrane translocase subunit TIM50; TIMM8B, mitochondrial import inner membrane translocase subunit Tim8 B; TOMM20, translocase of outer mitochondrial membrane 20 homolog; TOMM5, mitochondrial import receptor subunit TOM5 homolog; TOMM70, translocase of outer mitochondrial membrane protein 70; TRIM28, Transcription intermediary factor 1-beta (Fragment); TUBA4A, Tubulin alpha chain (Fragment).

**Supplemental figure 4.** **Influence of *APOE4* and HFD on carbohydrate-supported mitochondrial respiration in skeletal muscle.** Quadriceps collected at sacrifice at 8 months old after 4 months of diet was used to assess respiration in isolated mitochondria. Carbohydrate-stimulated mitochondrial oxygen consumption measured under basal (2 mM malate, 0.01 mM CoA, 2.5 mM carnitine, and 5 mM potassium pyruvate), state 3 (2.5 mM ADP), state 3 + glutamate (2 mM), and state 3S (10 mM succinate) conditions **(A)**. Coupling efficiency calculated from leak (basal) and state 3 respiration **(B).** H_2_O_2_/O_2_ flux ratio calculated from basal H_2_O_2_ and O_2_ flux **(C).** Carbohydrate-driven mitochondrial oxygen consumption measured during the creatine kinase (CK) clamp **(D)**. The slope of the relationship between ∆G_ATP_ and O_2_ flux was used to calculate respiratory conductance **(E)**. Sample size: respirometry data, n= 4-8/group; H_2_O_2_ data, n=3-6/group. Values are presented as mean ± SEM. LFD, low-fat diet; HFD, high-fat diet.

**Supplemental figure 5.** **Representative images of whole-tissue sections stained for fiber-typing.** Quadriceps collected at sacrifice at 8 months old after 4 months of diet was used to assess fiber-type distribution. Representative images of whole-tissue sections stained for muscle fiber membrane (laminin) and fiber types I, IIA, and IIB. Unstained fibers represent type IIX fibers. M, male; F, female; LFD, low-fat diet; HFD, high-fat diet; E3, apolipoprotein E3; E4, apolipoprotein E4.

**Supplemental figure 6. Lipid droplets in skeletal muscle.** Quadriceps collected at sacrifice at 8 months old after 4 months of diet was used to visually assess lipid droplet accumulation in serial tissue sections. Representative images are shown for whole-tissue sections stained for muscle fiber membranes (laminin) and lipid droplets (BODIPY 493/503) **(A)**. The localization of lipid droplets to specific fiber types was determined by staining first serial section for lipid droplets and second serial section for fiber-type **(B)**. Matching numbers shown for selected fibers repeated in both serial sections represent the same muscle fiber. M, male; F, female; LFD, low-fat diet; HFD, high-fat diet; E3, apolipoprotein E3; E4, apolipoprotein E4.
