# Supplementary figures and images for: APOE4 Exerts Partial Diet-dependent Effects on Energy Expenditure and Skeletal Muscle Mitochondrial Pathways in a Preclinical Model
Source: Function (Oxf). 2025 Mar 25;6(2):zqaf017. doi: 10.1093/function/zqaf017 (PMC11980864; doi:10.1093/function/zqaf017)

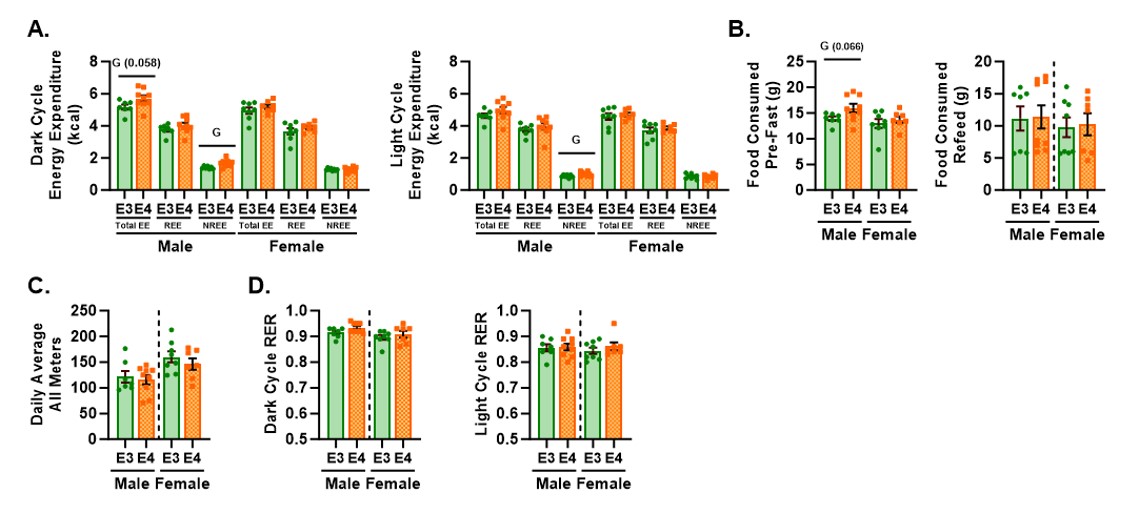

Supplement: zqaf017_Supplemental_Files [file zqaf017_supplemental_files.zip › Supplemental figure 1.jpg]

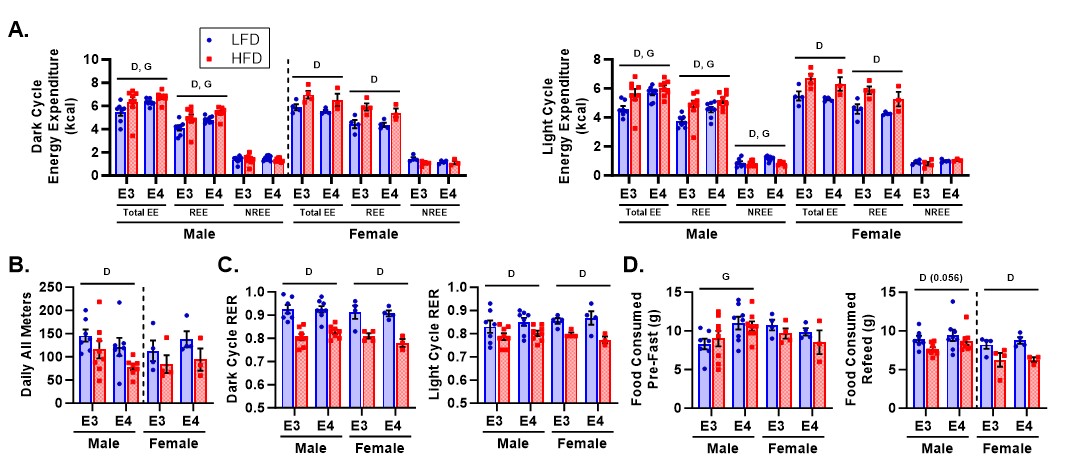

Supplement: zqaf017_Supplemental_Files [file zqaf017_supplemental_files.zip › Supplemental figure 2.jpg]

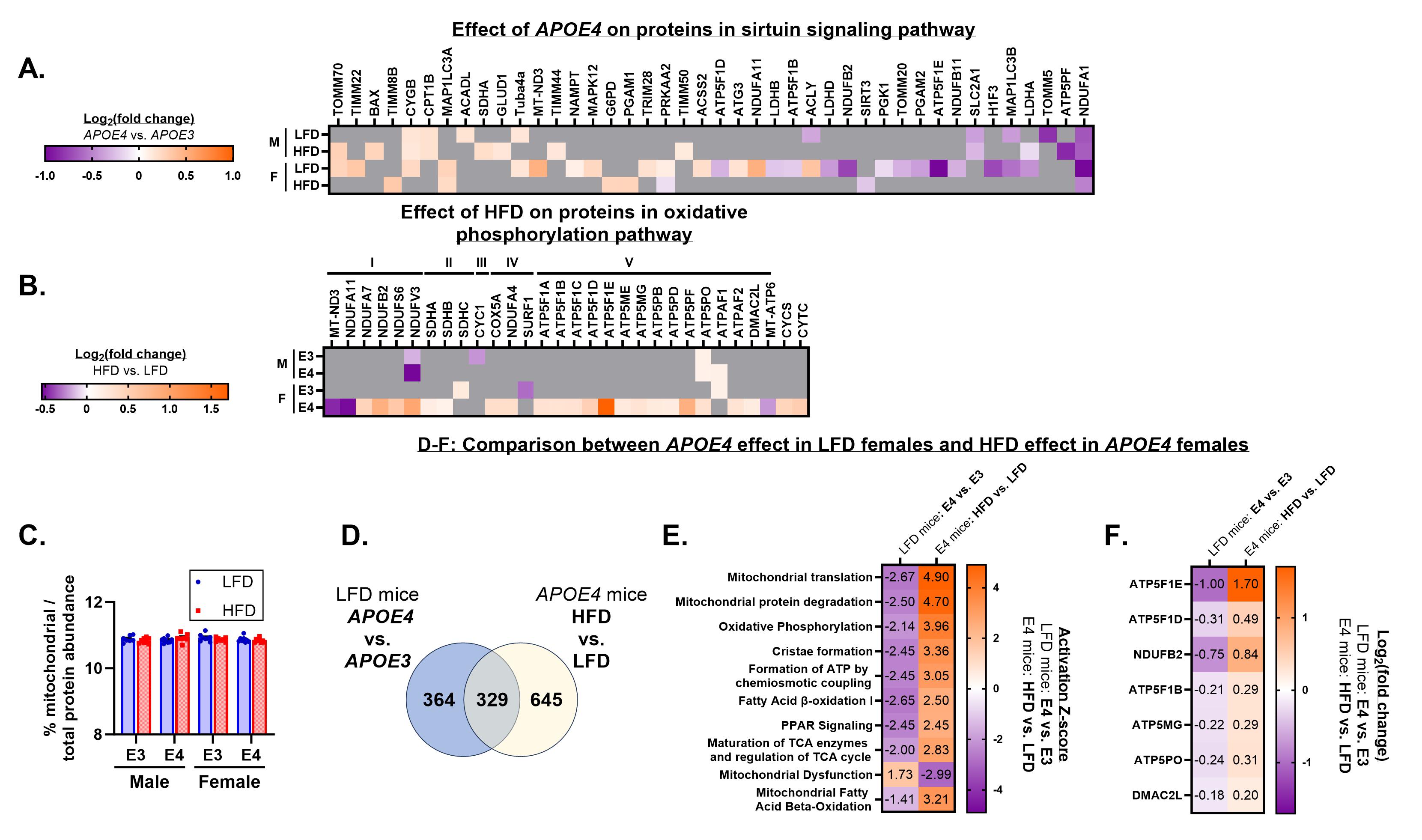

Supplement: zqaf017_Supplemental_Files [file zqaf017_supplemental_files.zip › Supplemental figure 3.jpg]

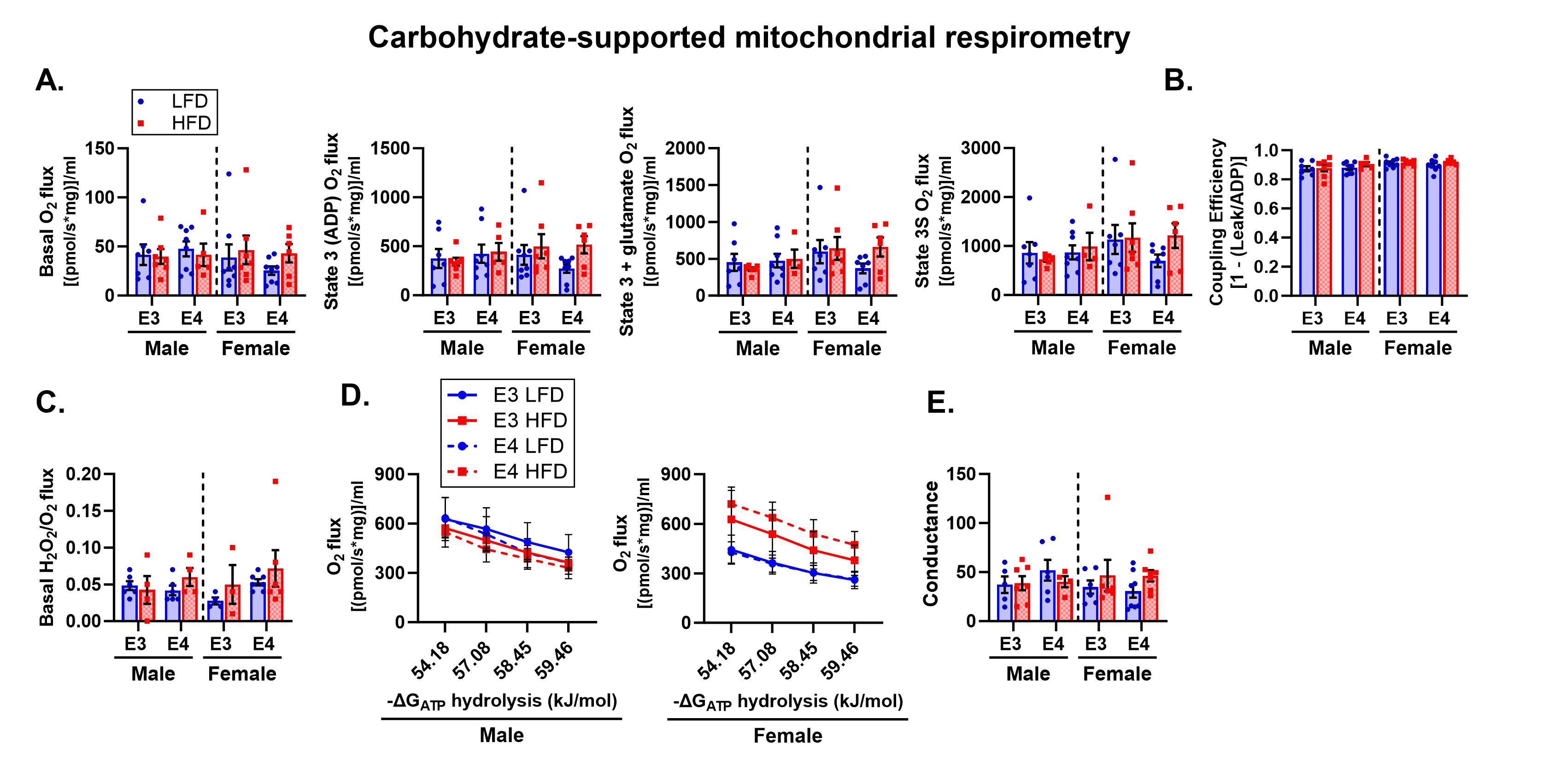

Supplement: zqaf017_Supplemental_Files [file zqaf017_supplemental_files.zip › Supplemental figure 4.jpg]

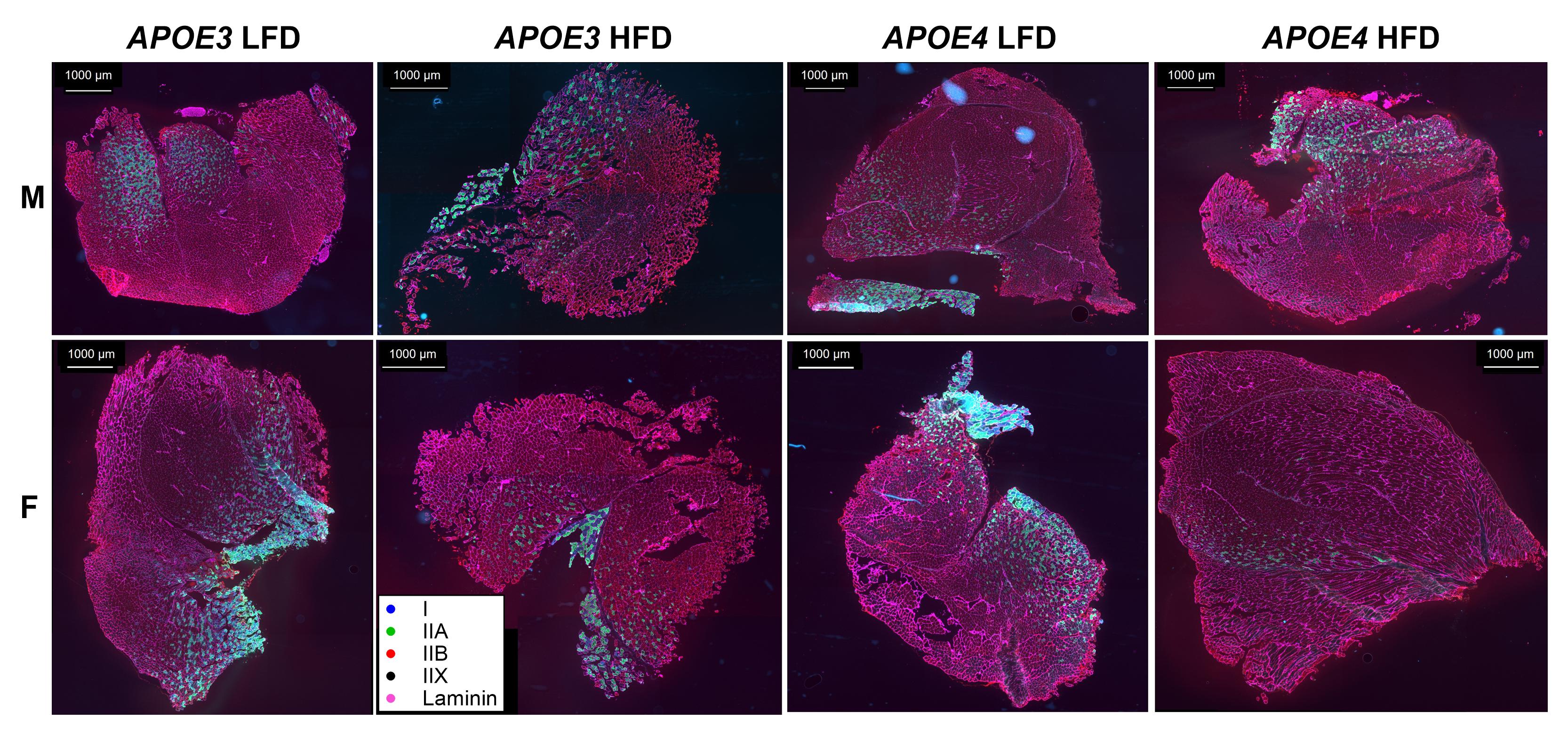

Supplement: zqaf017_Supplemental_Files [file zqaf017_supplemental_files.zip › Supplemental figure 5.jpg]

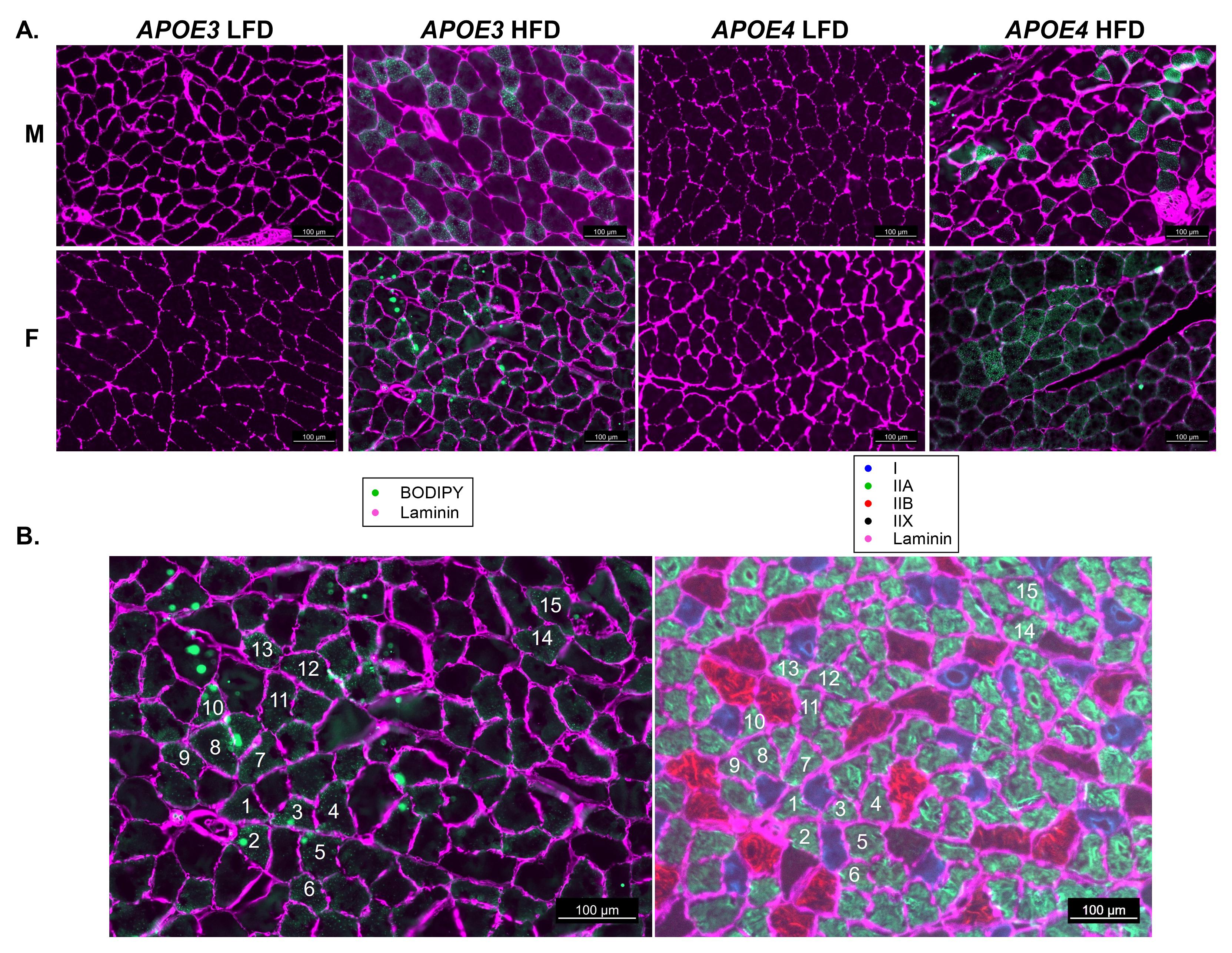

Supplement: zqaf017_Supplemental_Files [file zqaf017_supplemental_files.zip › Supplemental figure 6.jpg]
